# Characterization of *S-*glycosylated glycocins containing three disulfides

**DOI:** 10.1093/jimb/kuaf028

**Published:** 2025-09-08

**Authors:** Rachel M Martini, Chandrashekhar Padhi, Wilfred A van der Donk

**Affiliations:** Department of Biochemistry University of Illinois at Urbana-Champaign, Urbana, IL 61801, USA; Department of Chemistry and Howard Hughes Medical Institute, University of Illinois at Urbana-Champaign, Urbana, IL 61801, USA; Department of Biochemistry University of Illinois at Urbana-Champaign, Urbana, IL 61801, USA; Department of Chemistry and Howard Hughes Medical Institute, University of Illinois at Urbana-Champaign, Urbana, IL 61801, USA; Carl R. Woese Institute for Genomic Biology, University of Illinois at Urbana-Champaign, Urbana, IL 61801, USA

**Keywords:** RiPPs, leader peptidase, double Gly motif

## Abstract

Glycocins are a growing family of ribosomally synthesized and posttranslationally modified peptides (RiPPs) that are *O*- and/or *S-*glycosylated. Using a sequence similarity network of putative glycosyltransferases, the *thg* biosynthetic gene cluster (BGC) was identified in the genome of *Thermoanaerobacterium thermosaccharolyticum*. Heterologous expression in *Escherichia coli* showed that the glycosyltransferase (ThgS) encoded in the BGC adds *N-*acetyl-glucosamine (GlcNAc) to Ser and Cys residues of ThgA. The peptide derived from ThgA, which we name thermoglycocin, was structurally characterized and shown to resemble glycocin F. In addition to two nested disulfide bonds also present in glycocin F, thermoglycocin contains a third disulfide bond creating a C-terminal loop. Unexpectedly, ThgA lacks the common double glycine motif for leader peptide removal by a C39-peptidase. Based on AlphaFold3 modeling, we postulated that cleavage between the leader and core peptide would occur instead at a GK motif, which was experimentally confirmed for an orthologous BGC from *Ornithinibacillus bavariensis*. Its structurally similar product termed orniglycocin was also produced in *E. coli* and carries two GlcNAc moieties on two Cys residues. The C39 peptidase domain of the peptidase-containing ATP-binding cassette transporter (PCAT) from this BGC removed the leader peptide after a Gly-Lys motif and the orniglycocin so produced demonstrated antimicrobial activity. This study adds to the small number of characterized glycocins, employs AlphaFold3 to predict the leader peptide cleavage site, and suggests a common naming convention similar to that established for lanthipeptides.

**One-Sentence Summary**: Thermoglycocin from *Thermoanaerobacterium thermosaccharolyticum* and orniglycocin from *Ornithinibacillus bavariensis* were produced heterologously in *E. coli*, shown to contain three disulfide bonds and two GlcNAcylations, and were released by a unique C39 protease that cleaves at a Gly-Lys sequence.

## Graphical Abstract

**Graphical Abstract:**
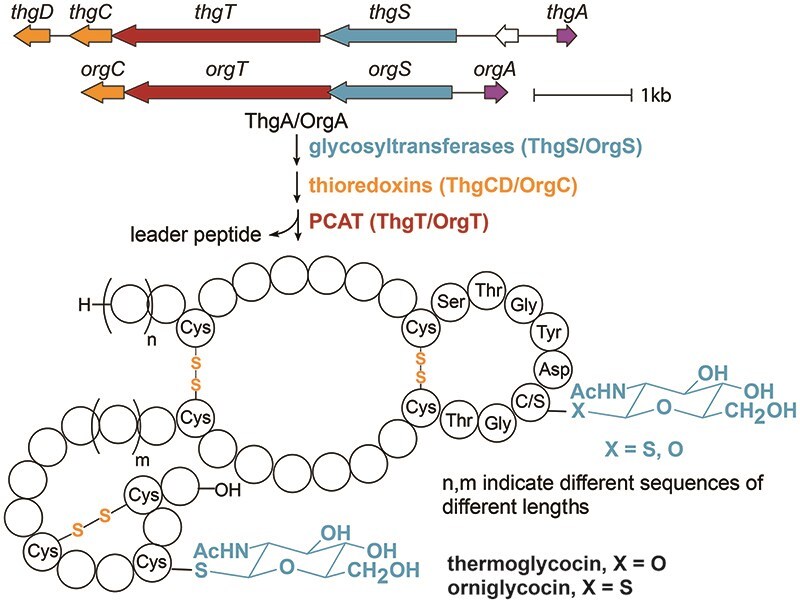

## Introduction

Recent investigations of ribosomally synthesized and posttranslationally modified peptides (RiPPs) have yielded many novel posttranslational modifications (Nguyen et al., 2024). RiPPs are synthesized as a precursor peptide that contains two regions: a leader peptide and a core peptide (Arnison et al., 2013,Oman & van der Donk, 2010). The core peptide is acted on by modifying enzymes encoded in the biosynthetic gene cluster (BGC), after which the leader peptide is typically cleaved off by proteolysis to create the mature peptide product (Eslami & van der Donk, 2023, Oman & van der Donk, 2010). Glycocins are a growing family of RiPPs, characterized by glycosylations on Ser, Thr, or Cys residues (Hata et al., 2010, Izquierdo et al., 2009, Kaunietis et al., 2019, Main et al., 2020, Maky et al., 2021, Maky et al., 2015, Nagar & Rao, 2017, Norris & Patchett, 2016, Oman et al., 2011, Ren et al., 2018, Stepper et al., 2011). Compared to *O*-linked glycosylations, *S*-linked glycosylations are rare in biology but they have greater stability and resistance to chemical and enzymatic cleavage (De Leon et al., 2017,Maynard et al., 2016). Previous studies have shown that glycosyltransferases from glycocin biosynthetic pathways can be used to glycosylate non-native substrates (Fujinami et al., 2021,Oman et al., 2011). Thus, the prevalence of *S*-linked glycosylations in glycocins opens up opportunities for the application of their glycosyltransferases to other fields (Sharma et al., 2021,Wang et al., 2014). Given the ubiquitous nature of glycosylations with *N-*acetyl-glucosamine (GlcNAc) in eukaryotic organisms (Hart et al., 2011), enzymes that generate *S*-linked GlcNAc modifications are particularly attractive for engineering purposes (Maynard et al., 2016). We therefore set out in this study to identify and characterize additional *S-*glycosyltransferases that would conjugate GlcNAc to Cys residues in peptides and to identify their products.

We used heterologous expression in *Escherichia coli* to produce and characterize two *O*/*S-*glycosylated peptides. A BGC (termed the *thg* locus) from the thermophilic bacterium *Thermoanaerobacterium thermosaccharolyticum* was chosen for investigation because enzymes from thermophiles generally display high stability that is desirable for engineering and use in biocatalytic processes (Chatterjee et al., 2023,Chettri et al., 2021,Gomes et al., 2016,Zhu et al., 2020). Indeed, other enzymes from this organism have been investigated for their potential use in biotechnology because of their thermostable and robust properties (Pei et al., 2012). In addition, a similar BGC from *Ornithinibacillus bavariensis* J43TS3 was selected for investigation (*org* locus).

In this work, we co-expressed the precursor peptides ThgA and OrgA with their glycosyltransferases ThgS and OrgS, respectively, in *E. coli* resulting in predominantly bisglycosylation with GlcNAc. The sites of glycosylation and the disulfide pattern of the products were determined through tandem mass spectrometry as well as proteolytic digest, confirming a disulfide pattern postulated previously (Norris & Patchett, 2016), which demonstrated that the products are members of the glycocin F family. Further, by reconstituting the activity of the C39-peptidase domain of the peptidase-containing ATP-binding cassette transporter (PCAT) in the *org* BGC, we showed that peptide maturation occurs by proteolytic cleavage at the non-canonical Gly-Lys motif instead of the well-studied double Gly motif, thus expanding the recognition site specificity of C39-peptidases and facilitating correct prediction of the final products. We show that the glycocin from *O. bavariensis* has antibiotic activity.

## Materials and Methods

### General Methods

Chemicals and media for cultures were purchased from Thermo Fisher Scientific or Sigma Aldrich unless otherwise stated. Polymerase chain reactions were carried out using a C1000 Bio-Rad thermocycler and were catalyzed using Q5 polymerase (NEB). Matrix-assisted laser desorption/ionization time-of-flight (MALDI-TOF) mass spectrometry analyses were carried out using a Bruker UltrafleXtreme instrument (Bruker Daltonics) through the UIUC Mass Spectrometry facility, using 50 mg/mL Super-DHB (Sigma, catalog number 50862-1G-F) dissolved in 80% acetonitrile and 0.1% trifluoroacetic acid (TFA) as matrix. MALDI-TOF mass spectra were obtained by desalting peptides using a C18 ZipTip, elution with 8 μL of 80% aqueous acetonitrile containing 0.1 % TFA, and then mixing 1:1 v/v with matrix before spotting on a MALDI plate.

### Identification of the *thg* BGC

A BLAST search (Boratyn et al., 2013) using the UniProt database (Consortium, 2024) with GccA as a query sequence, an E-value of 5 for the sequence retrieval, a maximum BLAST sequence number of 1,000, and an E-value of 5 for the sequence similarity edge calculation was used to generate a list of proteins related to this GlcNAc glycosyltransferase. The sequence similarity network (SSN) (Atkinson et al., 2009) webtool from the Enzyme Function Initiative-Enzyme Tools (EFI-EST) (Gerlt et al., 2015) was used to generate an SSN from the output of the BLAST search. The SSN was visualized using Cytoscape (Shannon et al., 2003) (Supplementary Fig. S1) with a filter value of 50%. The network was manually inspected for enzymes related to known glycosyltransferases involved in glycocin biosynthesis, especially those that resembled the GlcNAc *S-*glycosyltransferases AsmA (Main et al., 2020) and GccA (Stepper et al., 2011; Venugopal et al., 2011), to identify potential orthologs. To further narrow potential BGCs to likely glycocin BGCs, the EFI-Genome Neighborhood tool was used to identify glycosyltransferases with a PCAT (PFAM: PF03412) nearby using a neighborhood size of 10 and minimal co-occurrence percentage lower limit set to 20.

### Plasmid Assembly

Genes encoding ThgA, ThgS, OrgA, and OrgS, were codon-optimized for *E. coli* and ordered as double-stranded DNA from Twist Biosciences (see sequences in Table S1). In addition, genes encoding residues 33–157 of ThgT (truncated to aid in solubility), and the N-terminal 156 residues of OrgT containing the C39 peptidase domains from the PCATs of the respective BGCs were ordered codon-optimized. The synthetic genes were inserted into pRSFDuet-1 (*thgA* and *thgS*) or pACYCDuet-1 (*thgT33-157* and *thgS*) with HiFi Gibson assembly master mix (NEB). Similarly, *orgA* and *orgS* genes were cloned into the MCS1 and MCS2 of pRSFDuet-1 vector, respectively. The gene *orgT156* was cloned into a pET28a backbone. See Table S1 for the precise insertion sites into the plasmids. The assembled plasmids were used to transform chemically competent *E. coli* NEB Turbo cells that were then plated on LB agar with the appropriate antibiotic. Plasmid sequences were verified via Sanger DNA sequencing at the UIUC Core Sequencing Facility or using whole plasmid sequencing (Plasmidsauraus).

### Heterologous Expression of Peptides and Proteins and *in Vitro* Modification

Plasmids were used to transform *E. coli* Express SHuffle cells (NEB; for ThgA/ThgS/ThgT33-157) or *E. coli* BL21 (DE3) TUNER (for co-expression of ThgA/ThgS, or OrgA/OrgS), or *E. coli* BL21(DE3) T1^R^ competent cells (for expression of ThgS and MBP-ThgT33-157). *Escherichia coli* BL21 (DE3) TUNER cells were also used for the expression of recombinant OrgT156_._ A single colony was used to inoculate small (5 mL) cultures of LB containing 50 mg/L kanamycin (for pRSFDuet-1 and pET28), chloramphenicol (for pACYCDuet-1), and 100 mg/L spectinomycin (for SHuffle cells). Cultures were grown overnight at 37 °C, while shaking. For expression in *E. coli* BL21 (DE3) TUNER cells, a modified TB medium (Padhi et al., 2024) was used containing 24 g/L yeast extract, 20 g/L tryptone, and 2% glycerol supplied with 17 mM KH_2_PO_4_, and 72 mM K_2_HPO_4_ post-sterilization. The small cultures were diluted in 1 L of terrific broth (TB) and grown at 37 °C for TUNER cells (30 °C for SHuffle cells) to OD_600 _= 0.6. Cultures were incubated on ice for 20 min. Isopropyl ß-D-1-thiogalactopyranoside (IPTG) (GoldBio) was added to 0.5 mM. Cultures were grown overnight at 18 °C (16 °C for SHuffle cells). Cells were harvested via centrifugation at 8,000 x*g* for 10 min.

For proteins and peptides expressed in SHuffle cells, the harvested pellet was resuspended in lysis buffer (20 mM NaH_2_PO_4_ pH 7.5, 500 mM NaCl, 0.5 mM imidazole). Cells were lysed by sonication, and cell debris was removed via centrifugation at 45,000 x g for 1 hr. Supernatant was loaded onto a Ni-NTA immobilized metal affinity chromatography column (Takara Bio). Beads were washed with 20 mM NaH_2_PO_4_ pH 7.5, 500 mM NaCl, and 30 mM imidazole. Peptide or protein was eluted with 20 mM NaH_2_PO_4_ pH 7.5, 100 mM NaCl, 1 M imidazole. In the case of ThgS, the buffer was exchanged to protein storage buffer (50 mM HEPES pH 8, 300 mM NaCl, 10% glycerol) using an ultracentrifugal filter (Amicon) with a 30-kDa molecular weight cut off and frozen at −80 °C in small aliquots for later use. The buffer containing MBP-tagged ThgT33-157 purified by Ni-affinity chromatography was similarly exchanged to 50 mM Tris pH 8.

In case of peptides and proteins expressed in TUNER cells, the harvested pellet was resuspended in 50 mM Tris-HCl buffer containing 300 mM NaCl, 10% glycerol, and 20 mM imidazole at pH 8.0 (NPI_20_; 20 representing the imidazole concentration in mM) followed by cell lysis by sonication at an amplitude of 40% for 20 cycles of 5 s on/off with a 6-mm probe. The cell lysate was centrifuged at 12,000 × g to obtain the soluble fraction, which was then incubated with Ni-NTA agarose (MCLAB) for 1 hr at 4 ⁰C. The beads were subsequently washed with 3 × column volume (CV) of NPI_40_, followed by elution with 3 × CV of NPI_750_. Eluted peptides of proteins were exchanged into 50 mM Tris-HCl buffer containing 100 mM NaCl and 10% glycerol.

Modified or unmodified ThgA was further purified by preparative reversed-phase HPLC (Macherey-Nagel NUCLEODUR C18, 5 μm, 250×10 mm column) on an Agilent 1260 Infinity II HPLC system. Solvent A contained 0.1% TFA in H_2_O. Solvent B contained 0.1% TFA in acetonitrile. The gradient increased linearly from 2 to 60% B over 35 min. The yield of modified ThgA co-expressed with ThgS was about 150 μg per liter of culture.

### *In Vitro* Glycosylation of ThgA By ThgS

*In vitro* glycosylation of ThgA by ThgS was performed as described previously for other glycosyltransferases (Wang & van der Donk, 2011) using uridine-5’-diphosphate-α-D-glucose (UDP-Glc) or uridine-5’-diphosphate-α-D-*N*-acetylglucosamine (UDP-GlcNAc; Sigma) under reducing conditions (1 mM tris(2-carboxyethyl)phosphine, TCEP). The reaction was incubated overnight at 25 °C. Peptide was desalted using a C18 SPE column and lyophilized. To facilitate the formation of disulfide bonds in ThgA after glycosylation, the glycosylated peptide was incubated overnight with a mixture of oxidized and reduced glutathione as described previously (Wu et al., 2019).

Fully modified peptide (mThgA) was purified by reversed-phase HPLC using an Agilent 1260 Infinity analytical HPLC with a Vydac SelectaPore monomeric C18 column (90 Å, 5 µm, 2.1 mm ×150 mm). All mThgA-containing fractions were collected and lyophilized for storage at −20 °C.

### Identification and Characterization of Post-Translational Modifications

The identity of the sugar modification was determined by acid-catalyzed hydrolysis and gas chromatography-mass spectrometry (GC-MS) of the cleaved sugar. Sugar samples were converted into volatile derivatives as described previously (Oman et al., 2011), with the exception of a change in standards to *N*-acetylgalactosamine, *N*-acetylglucosamine, and *N*-acetylmannosamine. Chromatograms were acquired using a GC-MS system (Agilent Inc, CA, USA) consisting of an Agilent 7890 gas chromatograph, an Agilent 5975 MSD and a HP 7683B autosampler. Gas chromatography was performed on a ZB-5MS (60 m × 0.32 mm I.D. and 0.25-μm film thickness) capillary column (Phenomenex, CA, USA). The inlet and MS interface temperatures were 250 °C, and the ion source temperature was adjusted to 230 °C. An aliquot of 1 μL was injected with a split ratio of 10:1. The helium carrier gas was kept at a constant flow rate of 2 mL/min. The temperature program was: 5 min isothermal heating at 70 °C, followed by an oven temperature increase of 5 °C min^−1^ to 310 °C and a final 10 min at 310 °C. The mass spectrometer was operated in positive electron impact mode (EI) at 69.9 eV ionization energy at m/z 50–800 scan range combined with single ion monitoring (SIM) mode. For the SIM mode, a m/z 319 fragment for derivatized acetyl-hexosamines was analyzed (Mairinger et al., 2020) with 274 as a secondary qualifier fragment to minimize background from an interfering compound. Acquired peaks were evaluated by the Mass Hunter Quantitative Analysis B.08.00 (Agilent Inc., CA, USA) software. The instrument variability was within the standard acceptance limit (5%). GC-MS analysis was performed in the Carver Metabolomics Core (University of Illinois Urbana-Champaign Roy J. Carver Biotechnology Center).

LC-MS analysis was performed on an Agilent 1290 QToF for ESI-HR-MS and MS/MS analysis. For full length precursors, LC separation was conducted at 50 °C on a 5%-60% gradient of acetonitrile-water (+0.1% formic acid) over 8 min at 1 mL/min flow rate on a Phenomenex Aeris 3.6 μm WIDEPORE XB-C18 LC column (part nr. 00D-4482-E0). Mass spectra were collected in positive mode with 170 V fragmentor voltage at 10 spectra/s and 100 ms/spectrum. For digested peptide fragments, LC separation was conducted at 50 °C on a 10%–80% gradient of acetonitrile-water (+0.1% formic acid) over 11 min at 0.6 mL/min flow rate on a Phenomenex Aeris 2.6-μm PEPTIDE XB-C18 LC column (part nr. 00F-4505-E0). Mass spectra were collected in positive mode with 170-V fragmentor voltage at 10 spectra/s and 100 ms/spectrum. Tandem-MS fragmentation was achieved at mass-dependent normalized collision energies using a slope function [formula: (slope)×(m/z)/100 + offset value], where slope = 1 and offset = 20 and 30 for charge 3 and 2, respectively. HR-MS/MS analysis was performed using the Interactive Peptide Spectral Annotator (IPSA) tool (Brademan et al., 2019) and verified manually.

### mThgA and mOrgA Structural Characterization

To determine the location of GlcNAc modification, mThgA was labeled with NEM as described below and purified by C18 SPE column. The modified peptide was resuspended in buffer (100 mM Tris pH 8, 2 mM CaCl_2_) and digested with chymotrypsin (Worthington Biochemical Corporation) for 1 hr at 37 °C. Cleaved peptide was purified by C18 TopTip (Glycen) and then analyzed via liquid chromatography tandem mass spectrometry (LC-MS/MS) on an Agilent 1290 LC-MS QToF using a C18 column (Kinetex 2.6 μm) with a gradient of 2–60% B (for make-up, see above) over 8 min, a flow rate of 0.4 mL/min, and collision-induced dissociation (CID) energy of 30 eV.

The number of disulfide bonds in mThgA was determined by labeling with *N*-ethylmaleimide (NEM) under reducing conditions. The peptide was incubated at 70 °C for 15 min in buffer containing 100 mM sodium citrate (pH 6), 6 M guanidine HCl, 10 mM EDTA, and 10 mM TCEP. The reaction mixture was cooled to room temperature and NEM was added to 10 mM final concentration and the reaction was left in the dark for 30 min at 37 °C. The reaction mixture was desalted with a C18 ZipTip (Agilent) and analyzed by MALDI-TOF MS.

To determine the location of the disulfide bonds, monoglycosylated ThgA was resuspended in buffer (50 mM Tris pH 8, 0.5 mM CaCl_2_) and digested by thermolysin (Promega) with a 1:100 peptide to enzyme ratio for 1 hr at 37 °C. Digested fragments were desalted using C18 TopTip (Glycen) and lyophilized. LC-MS/MS was carried out using a C4 column (Jupiter, 5 μm, 300 Å pore size, 50×2 mm) with a gradient of 5–95% B (for make-up, see above) over 4 min, a flow rate of 0.4 mL/min, and CID energy of 30 eV. LysC digests of mThgA were carried out with an enzyme-to-substrate ratio of 1:20–1:100. The reaction was carried out in 20 mM Tris at pH 8 and incubated for 16 hr at room temperature.

For alkylation experiments of OrgS-modified OrgA (mOrgA), the samples were directly incubated with 5 mM TCEP at 50 °C for 30 min followed by incubation with 15 mM of freshly prepared NEM for 30 min in the dark at RT. For tandem-MS analysis, a desalted-alkylated mOrgA sample was dried under vacuum followed by resuspension in 50 mM Tris-HCl (pH 8) containing 100 mM NaCl. Chymotrypsin (Promega) or LysC (NEB) endoproteinase digestions were performed at 1:100 enzyme: substrate ratio for 2 hr in the presence of their respective buffers as per the manufacturer's recommendations, followed by incubation with 1 mM TCEP at 37 °C for 30 min. Samples were then analyzed by LC-MS/MS as described before for peptide fragment analysis.

### Characterization of the Protease Maturation At the Non-Canonical GK-motif

N-terminally 6xHis-tagged OrgT156 (OrgT156 encompassing the first 156 residues) was expressed in *E. coli* BL21 (DE3) TUNER cells and purified as described in the sections above. For activity assays, 50 µM of the substrate (mThgA or mOrgA produced in *E. coli*) was reacted with 5 µM of the enzyme (OrgT156) at room temperature for 16 hr followed by LC-MS analysis in the presence or absence of 1 mM TCEP. LC separation was conducted at 50 ⁰C on a 5%–60% gradient of acetonitrile-water (+0.1% formic acid) over 8 min at 1 mL/min flow rate on a Phenomenex Aeris 3.6 μm WIDEPORE XB-C18 LC column (part nr. 00D-4482-E0). Mass spectra were collected in positive mode with 170 V fragmentor voltage at 10 spectra/s and 100 ms/spectrum.

### Bioactivity Testing of Thermoglycocin

Bisglycosylated ThgA was cleaved with LysC to generate the core peptide, which was purified by HPLC. Fractions containing thermoglycosin as determined by MS were lyophilized and resuspended in sterile water to a final concentration of 10–100 μM. The peptide was screened for bioactivity against *Bacillus cereus* TZ417 (37 °C; ATCC medium 3: 3 g beef extract, 5 g peptone, 15 g agar per L), *E. faecalis* 29 212 (37 °C; ATCC medium 3), *G. thermodenitirificans* NM16-2 (50 °C; ATCC medium 3), *S. epidermis* (37 °C; BHI media), *Bacillus subtilis* ATCC 6633 (30 °C; LB), *B. subtilis* Δspβ (30 °C; LB), *Lactococcus lactis* (37 °C; TSB media), *B. megaterium* B-14308 (30 °C; ATCC medium 3), and *B. cereus* ATCC 14579 (30 °C; ATCC medium 3). Strains were grown overnight at the indicated temperature and in the medium shown. The cultures were diluted to a final OD of ∼0.05 in 20 mL molten agar media and poured over a plate prepared with set agar containing wells formed by placing a sterile 96 well plate over the agar while it set. Thermoglycocin (10 μL of 10–100 μM) was added to the wells, and plates were incubated overnight or until growth was visible in the top agar layer.

### Bioactivity Testing of Orniglycocin

The core peptide fragment of OrgT156-digested mOrgA (termed orniglycocin) was purified by HPLC, which resulted in 0.16 mg per liter of culture. The purified product was lyophilized and then dissolved in sterile water to a final concentration of 1 mM. The bioactivity of orniglycocin was tested for antibiotic potential against 10 strains, *B. subtilis* strain 168, *B. cereus* Z4222, *L. lactis* sp. cremoris NZ9000, *Bacillus licheniformis* NRRL NRS-1264, *Staphylococcus aureus* C5, *Staphylococcus simulans* 22, *Staphylococcus carnosus* TM300, *Micrococcus luteus* DSM 1790, and *Staphylococcus epidermidis* ATCC 12228. Overnight cultures were grown at 37 ⁰C in LB media followed by dilution to OD_600_ = 1. Base plates containing LB agar (1.8% agar w/v) were prepared and then overlayed with LB soft agar (0.5% agar w/v) containing 50 µL of the diluted cultures and allowed to dry. Wells were created using a 1 mL sterile tip and 2 μL of the resuspended orniglycocin was spotted in the wells. The plates were then incubated overnight at 37 ⁰C with subsequent imaging for zones of inhibition.

## Results and Discussion

### Identification of the *Thg* BGC

To identify potential new GlcNAc *S-*glycosyltransferases, we generated an SSN (Atkinson et al., 2009) from the output of a BLAST search with GccA as a query, using the EFI-EST version 2024_04/101 (Oberg et al., 2023). GccA is a glycosyltransferase encoded in the glycocin F gene cluster from *Lactobacillus plantarum* KW30 (Stepper et al., 2011; Venugopal et al., 2011). Since the leader peptides of all known glycocins are removed by a C39-like protease that is part of a bifunctional PCAT (Håvarstein et al., 1995), the Enzyme Function Initiative genome neighborhood tool (Oberg et al., 2023) was used to identify glycosyltransferases encoded near PCATs. Furthermore, we used comparisons to the sequences of the core peptides of known glycocin precursor peptides to identify systems that were predicted to make glycocins with different structures than previously reported family members. Based on these combined considerations and with the aim of characterizing a thermostable GlcNAc transferase, we chose a BGC from a thermophilic bacterium *T. thermosaccharolyticum* for further study and named it the *thg* BGC (for thermoglycocin as the final RiPP product after proteolytic procession).

**Fig. 1:**
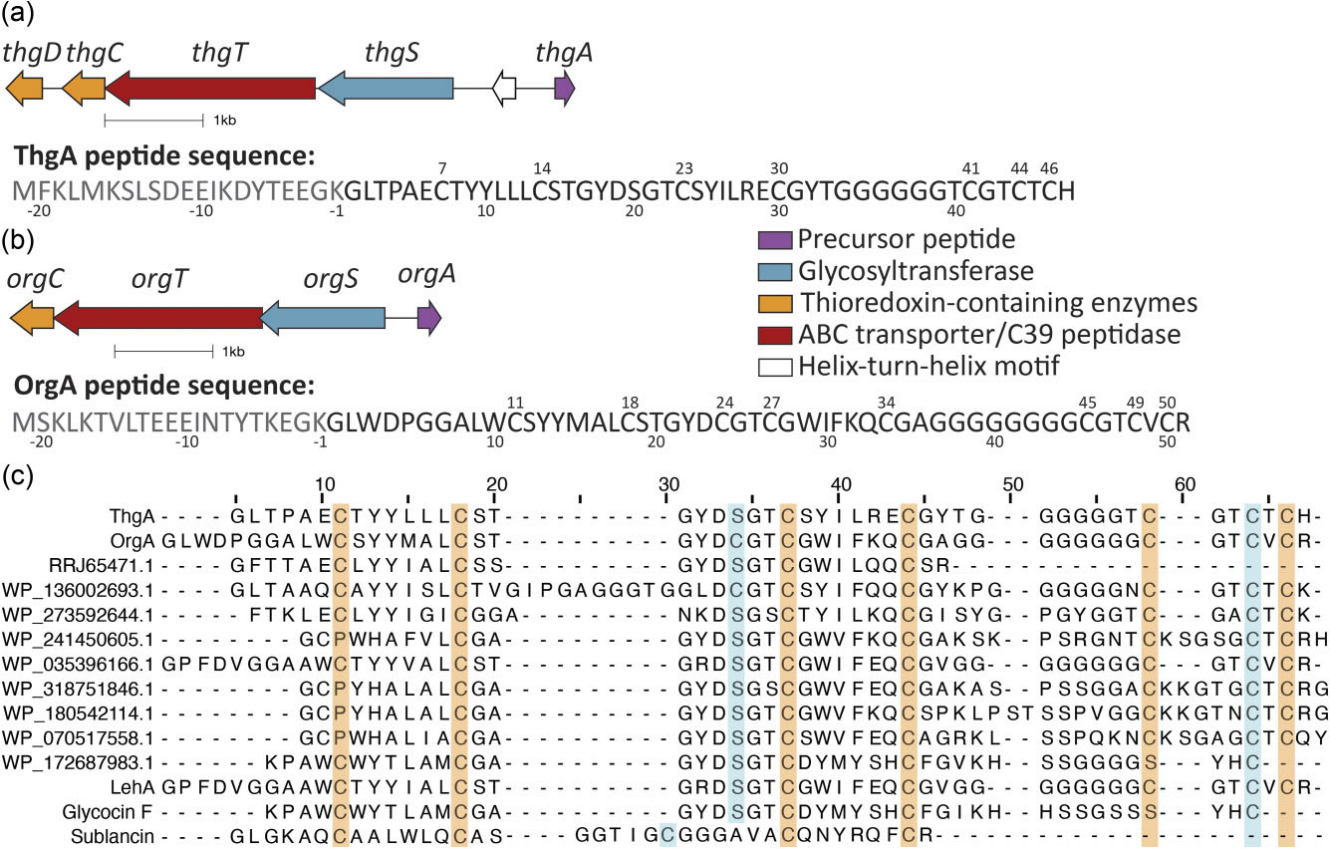
(a) Gene architecture of the *thg* BGC and sequence of the precursor peptide. The end of the leader peptide (residue − 1; leader peptide shown in grey) is putative and based on data in this study. Cys residues are numbered above the sequence. (b) Gene architecture of the *org* biosynthetic cluster in the genome of *O. bavariensis* J43TS3. The end of the leader peptide (shown in grey) is putative and based on data in this study. Cys residues are numbered above the sequence. (c) Alignment of the core peptides of ThgA and OrgA with related precursor peptides identified using NCBI BLAST and known glycopeptides, including two (LehA and OrgA previously named LehAvar) that were identified bioinformatically in a previous study (Norris & Patchett, 2016). LehA is encoded in the genome of *B. lehensis* G1 (AIC94358.1) and in the genome of *A. plakortidis* (KQL58965.1). LehAvar was found in cheese metagenome (accession ERX644124) (Norris & Patchett, 2016); OrgA is identical to LehAvar. The amino acids that align with glycosylated Ser/Cys residues in glycocin F, thermoglycocin and orniglycocin are highlighted in light blue, as is the Cys that is glycosylated in sublancin.

Using the nomenclature of the first glycocin BGC to be characterized (Dorenbos et al., 2002; Paik et al., 1998) responsible for producing sublancin (Oman et al., 2011), the proteins encoded in the *thg* cluster were given the names ThgA (precursor peptide), ThgS (glycosyltransferase), and ThgT (bifunctional protease transporter) (Fig. 1a). A pair of thioredoxin-like thiol-disulfide isomerases was annotated ThgC and ThgD. The precursor peptide with seven Cys residues is similar to a homologous peptide previously identified bioinformatically (Norris & Patchett, 2016) in the genomes of both *Bacillus lehensis* G1 (now *Shouchella lehensis* G1) (Noor et al., 2014) and *Alkalicoccobacillus plakortidis* DSM 19153 (Wang et al., 2016b) (Fig. 1b and c). An orthologous BGC was also identified in the genome of *O. bavariensis* J43TS3 (*org* cluster, Fig. 1b), which encodes a precursor peptide containing eight Cys residues in the predicted core region. An identical precursor peptide was previously reported in cheese metagenomic data (Norris & Patchett, 2016). The *thg* gene cluster also appears in the compilation of putative Type I glycocin BGCs reported by Singh & Rao (2021).

The ThgA precursor sequence is unique compared to previously experimentally characterized glycocins because of the presence of seven Cys residues in the predicted core peptide (Fig. 1a), whereas all previously experimentally characterized glycocins have five Cys residues (Hata et al., 2010; Izquierdo et al., 2009; Kaunietis et al., 2019; Main et al., 2020; Maky et al., 2021; Maky et al., 2015; Nagar & Rao, 2017; Norris & Patchett, 2016; Oman et al., 2011; Ren et al., 2018). The precursor ThgA is similar to OrgA, which contains eight Cys residues (Fig. 1b and c). The *org* BGC also encodes a glycosyltransferase (OrgS), a thioredoxin-like protein (OrgC), and a PCAT (OrgT).

### Characterization of the Glycocin Produced By the Thg BGC

**Fig. 2.**
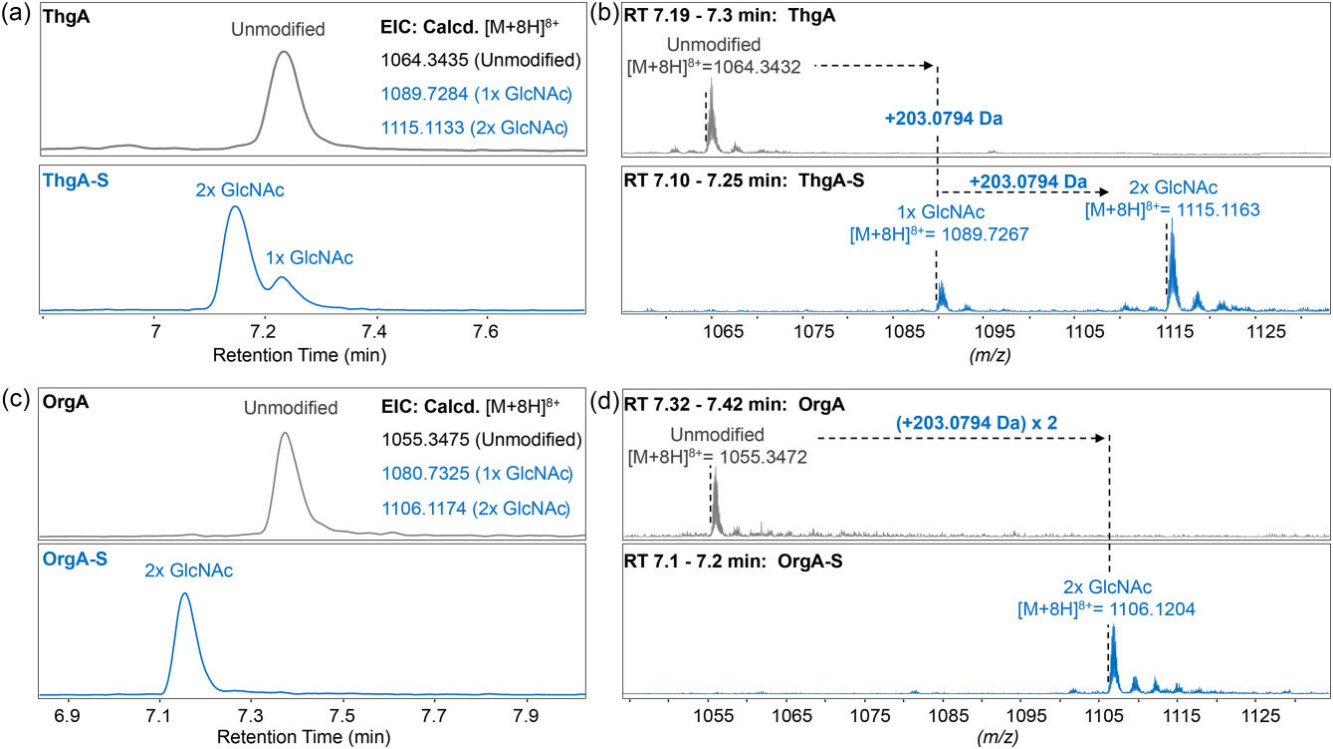
LC-ESI-HR-MS analysis of mThgA and mOrgA in the presence of TCEP. (a) Extracted ion chromatogram (EIC) of unmodified, monoGlcNAcylated, and bisGlcNAcylated ThgA after expression without (top) or with (bottom) ThgS (ThgA-S) and treatment with TCEP. Calculated m/z values are provided; see Table S1 for sequences. Deconvoluted [M + H]^+^ = 8507.6970 Da (unmodified), 8710.7764 Da (monoglycosylated) and 8913.8558 Da (bisglycosylated). (b) HR-MS spectra of ThgA (top) and mThgA (bottom) showing the observed masses and the mass shifts. (c) EIC of unmodified, monoGlcNAcylated, and bisGlcNAcylated full length OrgA after expression without (top) or with (bottom) OrgS (OrgA-S) and treatment with TCEP. Calculated EIC values are provided. Deconvoluted [M + H]^+^ = 8435.7297 Da (unmodified), 8638.8091 Da (monoglycosylated), and 8841.8885 Da (bisglycosylated). (d) HR-MS spectra of OrgA (top) and mOrgA (bottom) showing the observed masses and the mass shifts. Expressions were conducted in *E. coli* BL21 (DE3) TUNER cells. Both peptides showed near-complete conversion to bisglycosylated peptides.

Co-expression of N-terminally His_6_-tagged ThgA and ThgS in *E. coli* SHuffle T7 Express cells or *E. coli* BL21 (DE3) TUNER cells yielded two peptide products showing mass increases of 203.07 Da and 406.14 Da compared to the predicted mass of the unmodified precursor peptide (Fig. 2a) as determined by liquid chromatography-coupled electrospray ionization (ESI) high-resolution mass spectrometry (LC-ESI-HR-MS). These mass differences verified the addition of two *N*-acetylhexoses in modified ThgA (mThgA) (Fig. 2a, for tandem-MS see below). Whereas co-expression in SHuffle cells produced mostly monoglycosylated products, expression in TUNER cells produced mostly bisglycosylated products. Acid-catalyzed hydrolysis was used to cleave the sugars from the peptide followed by their derivatization as described previously (Oman et al., 2011). Gas chromatography monitored by mass spectrometry (GC-MS) and comparison with authentic standards derivatized in the same manner identified the sugar as GlcNAc (Supplementary Fig. S3).

### *In Vitro* Glycosylation By ThgS and Determination of Disulfide Pattern

**Fig. 3.**
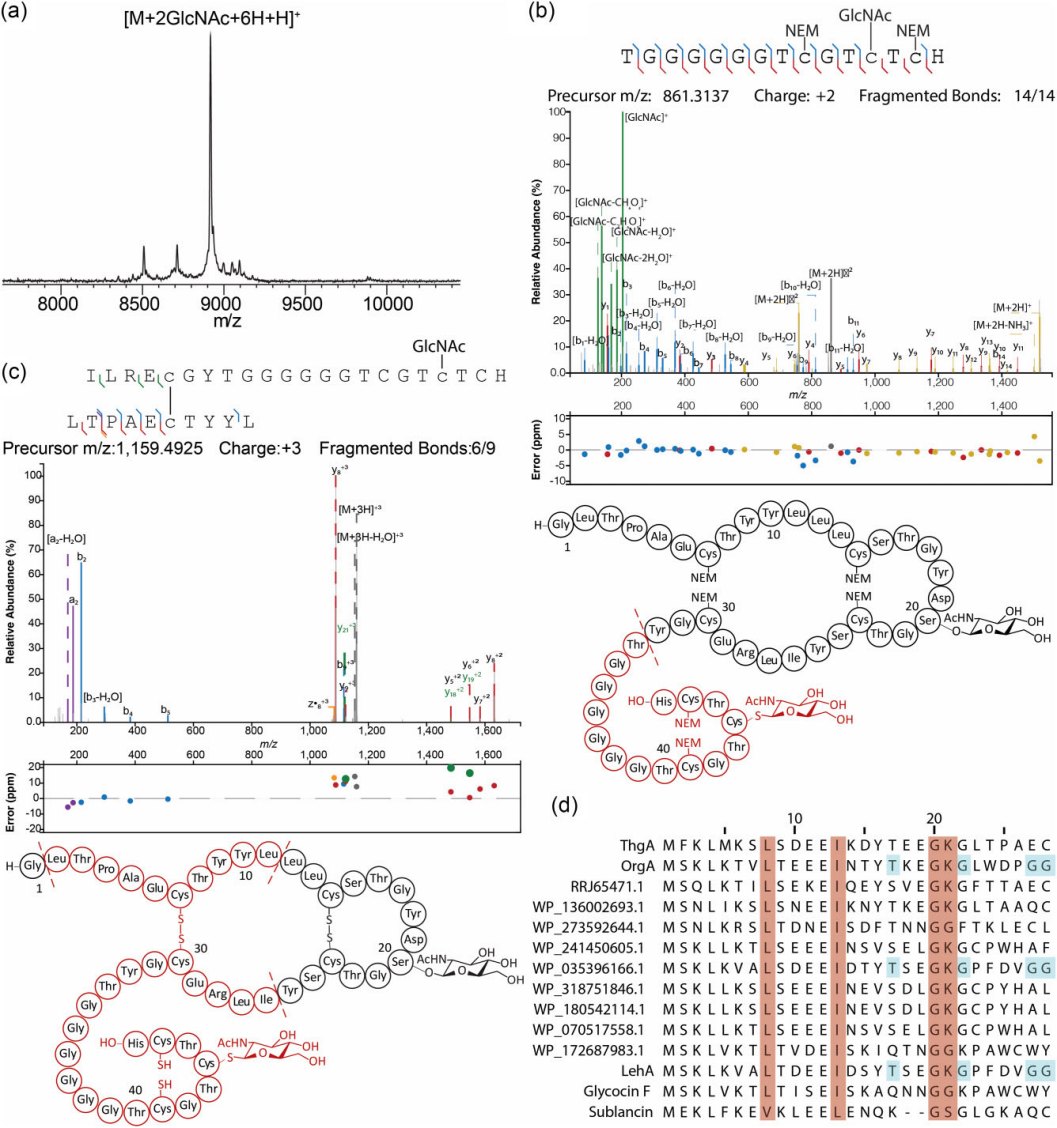
(a) MALDI-TOF mass spectrum of bisGlcNAcylated ThgA (observed mass: [M + H]^+^ = 8917.4, calculated average mass: [M + H]^+^ = 8919.4) after *in vitro* incubation with ThgS. Note that the three disulfides are reduced in the product (M+6H) because of the presence of TCEP in the enzymatic reaction, but some reoxidation prior to analysis may have occurred. (b) LC-MS/MS spectrum of residues 33–47 of bisGlcNAcylated ThgA, alkylated with NEM, and digested by chymotrypsin (observed mass: [M + 2H]^2+^ = 861.3147, calculated monoisotopic mass: [M + 2H]^2+^ = 861.3137, calculated molecular ion = 1720.6118). Fragmentation data are consistent with the + 203 modification being on Cys44. Cys41 and Cys46 were alkylated with NEM. The diagram shows the position of the proteolytic fragment (red) in thermoglycocin. (c) LC-MS/MS of ThgA residues 2–11 and 26–47 (observed mass: [M + 3H]^3+^ = 1159.4978, calculated monoisotopic mass: [M + 3H]^3+^ = 1159.4925, calculated molecular ion = 3475.4540) connected by a disulfide bond. The diagram shows the position of the proteolytic fragment (red) in thermoglycocin. (d) Multiple sequence alignment of ThgA with the leader peptide region of precursor peptides of related putative and known glycocins. The predicted cleavage sites are indicated in brown as well as the residues at positions −9 and −14 from the predicted cleavage site. The alternative double Gly motif in OrgA and the residues in positions −7 and −12 with respect to this alternative double Gly site are shown in light blue. Panels b and c were prepared using the Interactive Peptide Annotator Webtool (Brademan et al., 2019). Panel d prepared using MPI Bioinformatics Toolkit (Gabler et al., 2020).

We next turned to *in vitro* characterization of ThgS. His_6_-ThgA and His_6_-ThgS were expressed separately in *E. coli* SHuffle T7 Express cells and purified by nickel-affinity chromatography. *In vitro* reaction of His_6_-ThgA with His_6_-ThgS in the presence of UDP-GlcNAc as a sugar donor and TCEP to reduce any disulfides, which was previously shown to be required for glycosylation of sublancin (Oman et al., 2011), resulted in two glycosylations (Fig. 3a). This observation provided further support that fully glycosylated ThgA (mThgA) contains two GlcNAc modifications.

To localize the position of the modified residues, the free Cys residues were alkylated with NEM. The derivatized peptide was digested with chymotrypsin, and the fragments were analyzed by LC-MS/MS. Fragmentation by CID showed GlcNAc modification at Cys44 (Fig. 3b) near the C-terminus with Cys41 and Cys46 alkylated by NEM.

The second glycosylation site was more difficult to determine as the second sugar dissociated in the tandem MS experiments, suggesting that the second GlcNAc was *O*-linked, which is a more labile linkage (Drummond et al., 2021; Kaunietis et al., 2019; Main et al., 2020; Stepper et al., 2011). The digestion with chymotrypsin allowed us to narrow down the second glycosylation to a peptide spanning Asp19 through Tyr25 (Supplementary Fig. S4). Because of the lability of the GlcNAc during tandem MS analysis, we were not able to conclusively determine whether the site of *O-*glycosylation was Ser20 or Thr22, but based on sequence homology with the glycocins ASM1 and glycocin F (Fig. 1c) (Main et al., 2020; Stepper et al., 2011), the *O-*glycosylation is highly likely to occur on Ser20.

Further support for this hypothesis comes from experiments with OrgA (Fig. 1b). This peptide has high homology with ThgA including the six Cys residues that form disulfides and the Cys that is *S*-glycosylated, but the equivalent position to Ser20 in ThgA is occupied by a Cys residue in OrgA (Fig. 1c). Overexpression of OrgA with its glycosyltransferase OrgS in *E. coli* BL21 (DE3) TUNER cells resulted in near-complete conversion to a product carrying two GlcNAc molecules (Fig. 2c and 2d). The position of the sugars was determined to be at Cys24 and Cys48 by MS-MS analysis after digestion with endoproteinase LysC and chymotrypsin (Figs S5 and S6). A similar analysis on the NEM-alkylated mOrgA further confirmed that indeed the two Cys that are not involved in disulfides are glycosylated (Fig. S7).

**Fig. 4.**
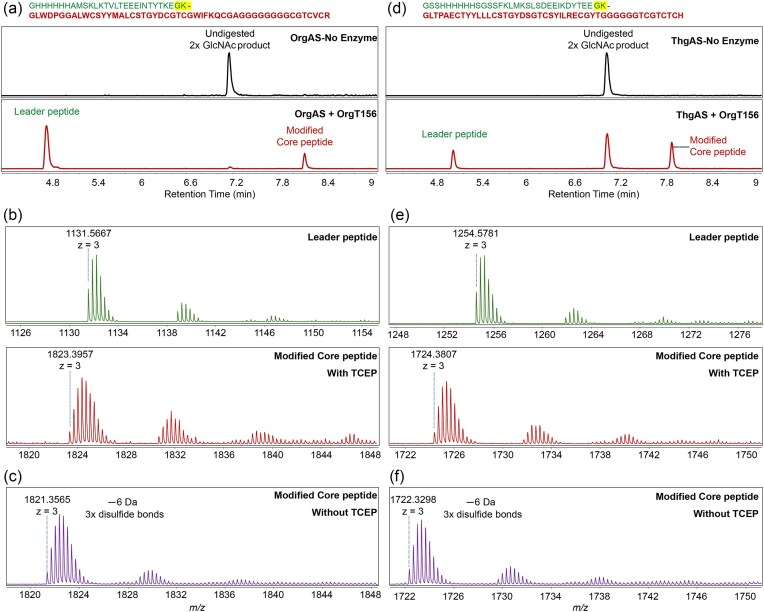
LC-ESI-HR-MS analysis of OrgT156-digested mOrgA and mThgA. (a) Extracted ion chromatogram (EIC) of mOrgA no-enzyme control (top) and OrgT156-digested mOrgA leader and core peptide fragments (bottom). (b) HR-MS spectra of the leader peptide fragment (top); deconvoluted [M + H]^+^ = 3392.6917 Da (theoretical), 3392.7001 Da (observed) for leader peptide and the core peptide fragment (bottom) of OrgT156-digested mOrgA in the presence of 1 mM TCEP. Deconvoluted [M + H]^+^ = 5468.1853 Da (theoretical), 5468.1871 Da (observed) for core peptide with bisglycosylation. (c) The mOrgA core peptide fragment in the absence of TCEP showing a 6 Da lower mass demonstrating the presence of three disulfide bridges formed in the final product. Deconvoluted [M + H]^+^ = 5462.166 Da (theoretical), 5462.0695 Da (observed) for core peptide with bisglycosylation. (d) EIC of mThgA no-enzyme control (top) and OrgT156-digested mThgA leader and core peptide fragments (bottom). (E) HR-MS spectra of the leader peptide fragment (top); deconvoluted [M + H]^+^ = 3761.7361 Da (theoretical), 3761.7343 Da (observed) for leader peptide and the core peptide fragment (bottom) of OrgT156-digested mThgA in the presence of 1 mM TCEP. Deconvoluted [M + H]^+^ = 5171.1376 Da (theoretical), 5171.1421 Da (observed) for core peptide with bisglycosylation. (f) The core peptide fragment generated in the absence of TCEP showing a 6 Da lower mass demonstrating that three disulfide bridges had formed in the final product. Deconvoluted [M + H]^+^ = 5165.1076 Da (theoretical), 5164.9894 Da (observed) for core peptide with bisglycosylation. The sequence of the full-length substrates used for the assays is shown above panels a and d highlighting the leader peptide in the first line and the core peptide inthe second line.

These data present strong but indirect support that ThgA is also bisglycosylated and that Ser20 carries one of the glycosylations. In turn, these findings suggest that ThgS is part of a growing class of glycosyltransferases able to catalyze both *O*- and *S*-linked glycosylations (Ahn et al., 2018; Main et al., 2020; Venugopal et al., 2011; Wang et al., 2014). All experimentally characterized glycocins have disulfide bonds that contribute to their remarkable stability and that play an important role in glycocin bioactivity (Bisset et al., 2018; Dorenbos et al., 2002). Reaction of mThgA isolated from *E. coli* with NEM in the presence of reductant resulted in addition of six NEM molecules (Supplementary Fig. S8) suggesting all six Cys residues that are not glycosylated are involved in disulfide bonding (see also discussion regarding Fig. 4 below that illustrates that mThgA contains three disulfides).

In *E. coli* (Fig. 2a) and *in vitro* (Fig. 3a), a minor product is a monoglycosylated peptide suggesting that the glycosylation is ordered. To interrogate which position is not glycosylated in the monoglycosylated product, the peptide was cleaved with thermolysin in the absence of reductant. Fragments were analyzed using LC-MS/MS. A triply charged ion of 1159.4978 Da was observed that corresponds to the mass of residues 2 to 11 of ThgA connected via a disulfide bond between Cys7 and Cys30 to a GlcNAcylated peptide consisting of residues 26 to 47 (Fig. 3c). Clean fragmentation was observed, and the fragment ions could be traced back to both peptides that were linked by a disulfide. Curiously, the fragment ions as well as the molecular parent ion suggest that the disulfide between Cys41 and Cys46 was not formed in this thermolysin digest peptide. While we do not have a good explanation for why this disulfide was not present, the data clearly show a disulfide between Cys7 and Cys30 and glycosylation of Cys44. Moreover, the thermolysin digest also resulted in an additional observed singly charged ion of 1467.5767 Da corresponding to residues 12–25 with a disulfide bond between Cys14 and Cys23 (Supplementary Fig. S9). These data suggest that Cys44 is glycosylated first in ThgA, with Ser20 being glycosylated second. These findings are also consistent with previous studies of the *O/S*-glycosyltransferase ThuS (Fujinami et al., 2021) that showed a preference for glycosylating Cys over Ser residues (Wang et al., 2014). This interpretation also explains why only bisglycosylated product was observed for OrgA (Fig. 2c). The full length bisglycosylated mThgA peptide was also analyzed by LC-MS/MS, showing minimal fragmentation between Lys at the −8 position and Tyr32 and no fragmentation between Cys41 and Cys46 (Fig. S10). As no or little fragment ions are expected within a ring structure, these observations together with the disulfides that exist between Cys41 and Cys46 (Fig. 3b) and Cys 14 and 23 (Supplementary Fig. S9) strongly suggest that thermoglycocin has the same nested disulfide bonding pattern of sublancin and glycocin F with an additional disulfide at the C-terminus (Fig. 3c). The protease digest and fragmentation patterns of mOrgA further support this conclusion (Supplementary Fig. S5). This pattern of disulfides was previously predicted for some of the peptides in Fig. 1c (Norris & Patchett, 2016).

### Prediction of the Leader Peptide Cleavage Site

Almost all RiPPs are made from a precursor peptide that contains an N-terminal leader peptide that is removed in a late biosynthetic step by a protease (Eslami & van der Donk, 2023; Montalbán-López et al., 2021). The responsible protease is often encoded within the BGC, and indeed the *thg* and *org* BGCs encode an ATP-dependent transporter with a C39-type N-terminal protease (ThgT and OrgT, Fig. 1). These bifunctional PCATs are frequently found in BGCs of a wide variety of RiPPs (Eslami & van der Donk, 2023; Håvarstein et al., 1995; Padhi et al., 2024) and their leader peptides are the most common type of leader peptide in RiPP biosynthesis (Montalbán-López et al., 2021). All currently characterized PCATs have been shown to cleave after GG, GA, or GS motifs, typically called the double glycine motif (Bobeica et al., 2019; Dirix et al., 2004a; Dirix et al., 2004b; Ishii et al., 2010). Given the lack of such a motif in ThgA in the predicted leader peptide (Fig. 1a), it was not clear where the leader peptide removal site is in ThgA. We made a multiple sequence alignment of the N-terminal part of ThgA with related peptides retrieved from the NCBI database using BLAST (Boratyn et al., 2013) (Fig. 3d). The structure of glycocin F (Stepper et al., 2011; Venugopal et al., 2011) suggests that its precursor peptide GccF is cleaved at a double glycine motif by the protease/transporter GccB. The double Gly motif of GccF (GG-K) corresponds to the sequence GK-G in ThgA, which was also hypothesized to be the leader peptide removal site in the glycocin Hyp1 (Fig. 3d) (Kaunietis et al., 2019). However, this predicted proteolytic site is unusual for a number of reasons. First, previously studied C39 peptidases were shown to be unable to accept charged residues in the −1 position (Furgerson Ihnken et al., 2008). Second, most PCAT substrates have hydrophobic residues (Leu, Val, Ile, Met) in positions −7 and −12 from the cleavage site, which occupy hydrophobic pockets in the PCAT as shown by X-ray crystallography (Bobeica et al., 2019). An example is the precursor peptide sequence to the glycocin sublancin (Fig. 3d). But for all the precursor peptides other than sublancin shown in Fig. 3d, the residues at position −7 from the putative G(−2)K(−1)-G(+1) cleavage site are hydrophilic (Asp/Glu/Ser/Thr/Asn/Lys) and at position −12 mostly negatively charged (Glu/Asp). We noted that the residues at positions −9 and −14 in these peptides are invariably hydrophobic (Leu, Ile, Val, Fig. 3d), possibly suggesting that the register has shifted in this set of peptides.

To investigate this hypothesis, we used AlphaFold3 (Abramson et al., 2024) modeling of the ThgA leader peptide and the protease domain of ThgT encompassing the N-terminal 157 residues (ThgT1-157, Supplementary Fig. S11). The model showed that residues Ile −9 and Leu −14 of ThgA fit into a hydrophobic groove on ThgT157 and that Lys −1 aligns with the catalytic Cys in the active site of the peptidase (Bobeica et al., 2019; Chen et al., 2001; Ishii et al., 2010). Thus, it seems that a group of PCATs that putatively cleave substrates after a GK sequence are able to do so by setting a different register in which now hydrophobic residues at positions −9 and −14 occupy the same pockets that normally are occupied by residues at positions −7 and −12. The known cleavage site to release glycocin F provides indirect support for such a change in register. The precursor peptide for this glycocin has the canonical double Gly motif, but it also has the hydrophobic residues in positions −9 and −14 and not −7 and −12 (Fig. 3d).

In an attempt to experimentally verify these predictions, the N-terminal C39 protease domain of ThgT was expressed in *E. coli* as has been done previously for the orthologous enzymes CvaB (Wu & Tai, 2004), ComA (Ishii et al., 2006), LahT (Bobeica et al., 2019), LctT (Furgerson Ihnken et al., 2008), BovT (Wang et al., 2016a), ColT (Wang et al., 2025), McyT (Wang et al., 2023a), and MfuT (Wang et al., 2023b). Unfortunately, a His_6_-tagged version was obtained in insoluble form under a variety of expression conditions. A maltose binding protein (MBP) fusion was successfully expressed and purified but did not show any proteolytic activity.

### Reconstitution of the C39 Protease Domain of OrgT

With the inability to experimentally confirm the predictions regarding the leader peptide cleavage site for thermoglycocin, we turned to the *org* BGC. OrgA contains both a GG and a GK motif in its putative leader peptide region (Fig. 3d). Our prediction was that OrgT would cleave at the latter since it would position two hydrophobic residues at positions −9 (Ile) and −14 (Leu). Conversely, if cleavage were to take place at the GG sequence, the residues at positions −9 and −14 would be Gly and Thr, respectively, or if the recognition follows the canonical C39 cleavage pattern the residues at positions −7 and −12 from the GG sequence would (coincidentally) again be Gly and Thr, respectively (Fig. 3d). Neither conforms with current knowledge about these enzymes. Based on a predicted structure by AlphaFold3 (Supplementary Fig. S12), the N-terminal 156 residues of OrgT were expressed in *E. coli* TUNER cells with an N-terminal His_6_-tag. OrgT156 was purified and reacted with mOrgA that had been modified with two GlcNAc groups by OrgS. Two products were observed by ESI-MS that unambiguously confirm that cleavage takes place after the GK motif (Fig. 4a and b) and that the product obtained in *E. coli* contains three disulfides (Fig. 4c). OrgT156 was also reacted with mThgA, which also resulted in cleavage at the GK site, albeit with lower efficiency (Fig. 4d–f). These data thus identify the first group of PCATs that do not cleave at a canonical double Gly site and for which the recognition relies on hydrophobic residues at positions −9 and −14. These data also show that the predicted glycocin from the *org* BGC has a much longer N-terminus than what would have been predicted based on cleavage at a double Gly site. The discovery of different cleavage sites for PCATs will be important for predicting the junction between leader and core peptides in genome mining studies.

### Bioactivity Testing of Thermoglycocin and Orniglycocin

**Fig. 5.**
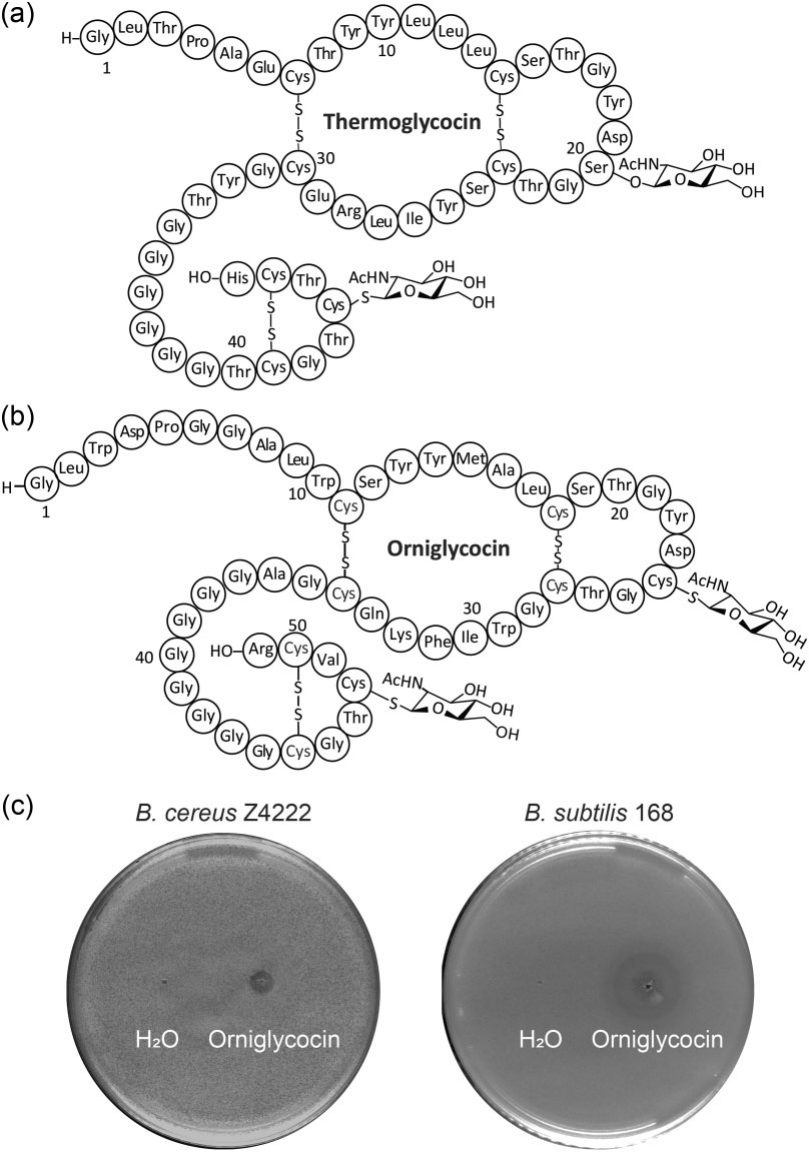
Structures of (a) thermoglycocin and (b) orniglycocin. (c) Bioactivity assay of orniglycocin. Zones of growth inhibition by orniglycocin that was spotted on an overlay of *B. cereus* Z4222 and *B. subtilis* strain 168. Sterile water was used as solvent for orniglycocin as well as for the control spot.

The data obtained in this study suggest a similar topology for the glycocins from *T. thermosaccharolyticum* and *O. bavariensis* (Fig. 5a) as the previously characterized compounds sublancin and glycocin F. Orniglycocin was tested for antimicrobial activity using agar diffusion assays against *B. subtilis* 168, *B. cereus* Z4222, *L. lactis* sp. cremoris NZ9000, *B. licheniformis* NRRL NRS-1264, *S. aureus* C5, *S. simulans* 22, *S. carnosus* TM300, *M. luteus* DSM 1790, and *S. epidermidis* ATCC 12228. Orniglycocin showed antibiotic activity against *B. cereus* Z4222 and *B. subtilis* 168 (Fig. 5b). Thermoglycocin was also tested but no antimicrobial activity was observed, possibly because the compound selectively targets organisms that co-habitat the environment of *T. thermosaccharolyticum*, an anaerobic thermophile, just as the glycocin pallidocin from the thermophile *Aeribacillus pallidus* targets other thermophilic bacteria (Kaunietis et al., 2019). An alternative explanation is that the bisGlcNAcylated compound produced in *E. coli* and cleaved by OrgT156 is different in structure as the native compound. We consider this explanation less likely given the similarity to orniglycocin as well as other glycocins.

### Suggested Nomenclature for Glycocin Biosynthetic Enzymes

Rapid growth in the field of glycocins suggests that a common naming convention for glycocin biosynthetic machinery may be desirable. A standardized nomenclature using the prefix Lan was proposed more than 30 years ago for the largest class of RiPPs, the lanthipeptides (de Vos et al., 1991), which has proven to be very useful in discussing lanthipeptide families, genes, and enzymes (Repka et al., 2017). For glycocins, because some BGCs were annotated before the protein function was known and for other BGCs genes were annotated in alphabetical order of their appearance in the BGC, the function of a certain protein cannot be immediately gleaned from its name. For instance, for three representative glycocins, sublancin, glycocin F, and enterocin F4-9, the protein naming is diverse: substrates are SunA, GccF, and Enf49A, glycosyltransferases are SunS, GccA, and EnfC, thiol-disulfide oxidoreductases are BdbAB, GccCD, and EnfB, and transporters/protease are SunT, GccB, and EnfT. We propose a uniform nomenclature for the biosynthetic enzymes of glycocins that may be identified in future studies that will immediately associate function with the name. The proposed nomenclature is a hybrid of the original naming scheme for sublancin (Oman et al., 2011; Paik et al., 1998) and glycocin F (Ahn et al., 2018; Stepper et al., 2011), the first two characterized glycocins. In the proposed nomenclature, the biosynthetic genes are designated with a generic locus symbol *gyc*, with a more specific genotypic designation for each glycocin member (e.g. *sun* for sublancin and *gcc* for glycocin F). The glycocin precursor peptides would be referred to using the generic term GycA, glycosyltransferases as GycS, peptidases as GycT, and disulfide isomerases as GycC and GycD. We used this scheme here for the naming of the *thg* and *org* BGCs.

## Conclusions

Thermoglycocin is the first characterized glycocin derived from an anaerobic thermophile. Our genome mining studies also uncovered orniglycocin as another example of an *S*-linked glycopeptide with antibacterial activity. Both orniglycocin and thermoglycocin contain three disulfide linkages and two GlcNAcylations. They differ in the glycosylation of a loop between two α-helices. The sequence of this eight amino acid loop is near identical, differing only in having a Ser in thermoglycocin and a cysteine in orniglycocin. Interestingly, these two differing residues are GlcNAcylated during their biosynthesis. In the final maturation step, novel C39-peptidases process both modified OrgA and ThgA to furnish orniglycocin and thermoglycocin by removing a leader peptide at a non-canonical Gly-Lys motif. As others have predicted based on genome sequences (Palaniappan et al., 2020; Singh & Rao, 2021), this study shows that the diversity of glycocin structures will continue to grow as more are characterized.

## Supplementary Material

kuaf028_Supplemental_File

## Data Availability

All data are incorporated into the article and its online Supplementary Material. Primary data are deposited at: Martini, Rachel; Padhi, Chandrashekhar; van der Donk, Wilfred (2025), ‘Characterization of antimicrobial S-glycosylated glycocins containing three disulfides’, Mendeley Data, V1, doi: 10.17632/m7zm6kk295.1
