# Supplementary material for: Characterization of S-glycosylated glycocins containing three disulfides
Source: J Ind Microbiol Biotechnol. 2025 Sep 8;52:kuaf028. doi: 10.1093/jimb/kuaf028 (PMC12457901; doi:10.1093/jimb/kuaf028)
Supplement: kuaf028_Supplemental_File [file kuaf028_supplemental_file.pdf]

Supplementary Information

Characterization of *S*-glycosylated glycocins containing three disulfides

Rachel M. Martini,[1,§] Chandrashekhar Padhi,[2,§] and Wilfred A. van der Donk[1-3,*]

[1] Department of Biochemistry University of Illinois at Urbana-Champaign, Urbana, Illinois 61801, USA.

[2] Department of Chemistry and Howard Hughes Medical Institute, University of Illinois at Urbana-Champaign, Urbana, Illinois 61801, USA.

[3] Carl R. Woese Institute for Genomic Biology, University of Illinois at Urbana-Champaign, Urbana, Illinois 61801, USA.

*Correspondence to: vddonk@illinois.edu

[§] These authors contributed equally to this study

TABLE OF CONTENTS:

SUPPLEMENTARY FIG. S1. SEQUENCE SIMILARITY NETWORK OF THGS RELATED PROTEINS. 3
SUPPLEMENTARY FIG. S2. SDS-PAGE GEL OF PURIFIED PEPTIDES AND PROTEINS. 4
SUPPLEMENTARY FIG. S3. IDENTIFICATION OF THGA GLYCOSYLATION USING GC-MS. 5
SUPPLEMENTARY FIG. S4. CHYMOTRYPSIN DIGEST FRAGMENT OF MTHGA. 6
SUPPLEMENTARY FIG. S5. ESI-HR-MS ANALYSIS OF LYSC AND CHYMOTRYPSIN-DIGESTED MORGA 7
SUPPLEMENTARY FIG. S6. ESI-HR-MS/MS OF LYSC AND CHYMOTRYPSIN-DIGESTED MORGA 8
SUPPLEMENTARY FIG. S7. ESI-HR-MS/MS OF NEM-ALKYLATED, CHYMOTRYPSIN-DIGESTED MORGA 9
SUPPLEMENTARY FIG. S8. NEM ALKYLATION OF MTHGA. 10
SUPPLEMENTARY FIG. S9. LC MS/MS DATA SUGGESTING A DISULFIDE BOND BETWEEN CYS14 AND CYS23 OF THGA. 11
SUPPLEMENTARY FIG. S10. LC-MS/MS OF THGA SHOWING LESS FRAGMENTATION IN PROPOSED DISULFIDE BONDED AREAS 12
SUPPLEMENTARY FIG. S11. THGA/THGT157 PREDICTED STRUCTURE. 13
SUPPLEMENTARY FIG. S12. PREDICTED STRUCTURE OF THE ORGT156/ORGA COMPLEX. 14
SUPPLEMENTARY FIG. S13. UHPLC TRACE OF FULLY MODIFIED PEPTIDES. 15
TABLE S1. PLASMIDS AND GBLOCKS USED IN THIS STUDY 16
TABLE S2. FRAGMENT IONS OBSERVED IN FIG. 3B. 20
TABLE S3. FRAGMENTS OBSERVED IN FIGURE 3C 22
TABLE S4. FRAGMENT IONS OBSERVED IN FIG S4. 23
TABLE S5. HR-MS/MS TABLE FOR CHYMOTRYPSIN-DIGESTED MODIFIED ORGA 24
TABLE S6. HR-MS/MS TABLE FOR LYSC-DIGESTED MODIFIED ORGA 25
TABLE S7. HR-MS/MS TABLE FOR CHYMOTRYPSIN-DIGESTED NEM-ALKYLATED MODIFIED ORGA 26
TABLE S8. HR-MS/MS TABLE FOR CHYMOTRYPSIN-DIGESTED NEM-ALKYLATED MODIFIED ORGA 27
TABLE S9. MS/MS FRAGMENTS SHOWN IN FIGURE S10 28

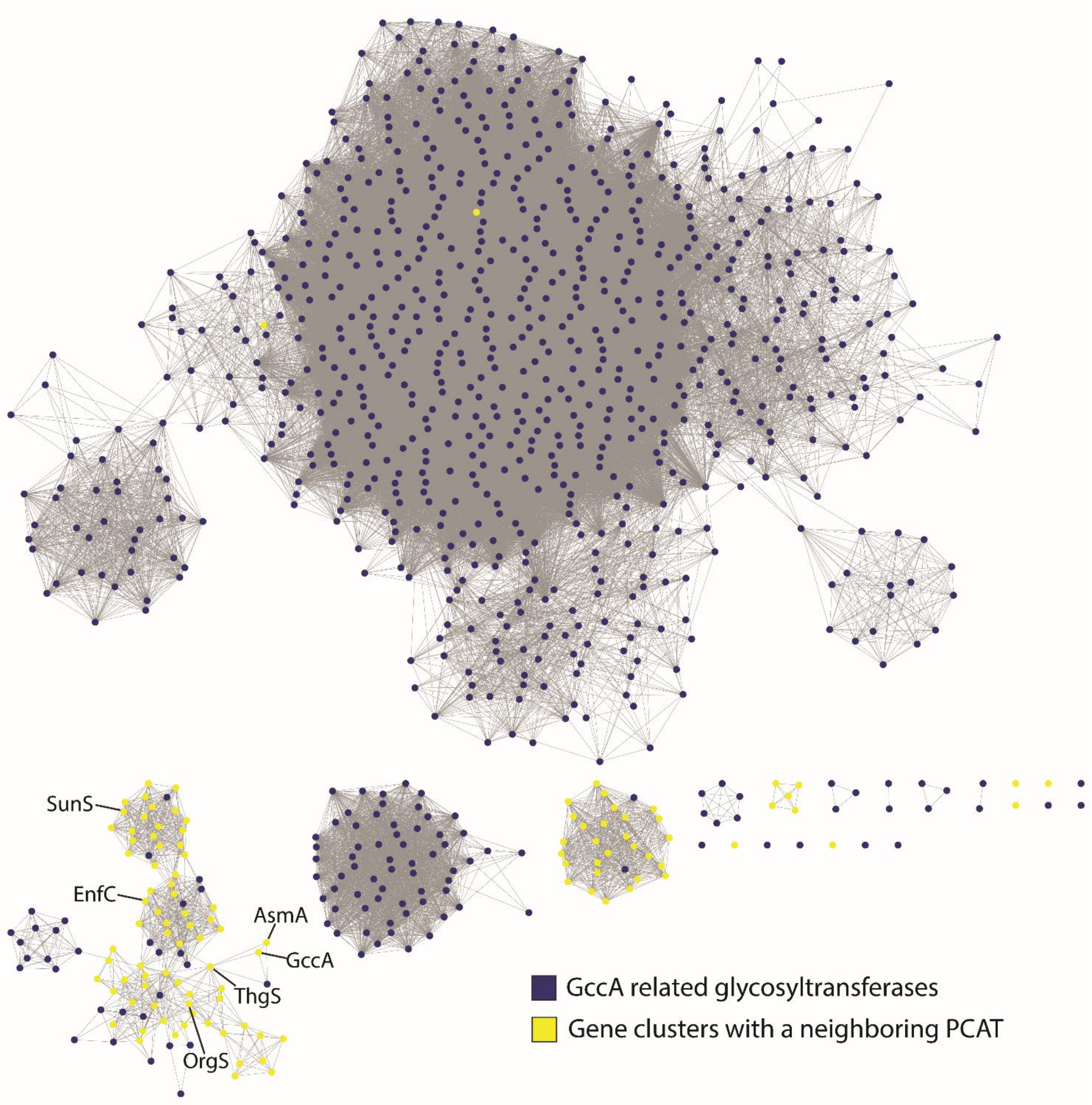

Supplementary Fig. S1. Sequence similarity network of ThgS related proteins.

Sequence similarity network (alignment threshold of 50) of proteins related to ThgS generated from UniProt database (Consortium, 2024). Nodes highlighted in yellow show proteins with a neighboring (within 10 genes) C39 peptidase (PFAM PF03412) determined by gene neighborhood network analysis (Oberg et al., 2023). Known glycosyltransferases involved in glycocin biosynthesis are labeled, including AsmA (Main et al., 2020), GccA (Stepper et al., 2011), EnfC (Maky et al., 2015), and SunS (Oman et al., 2011).

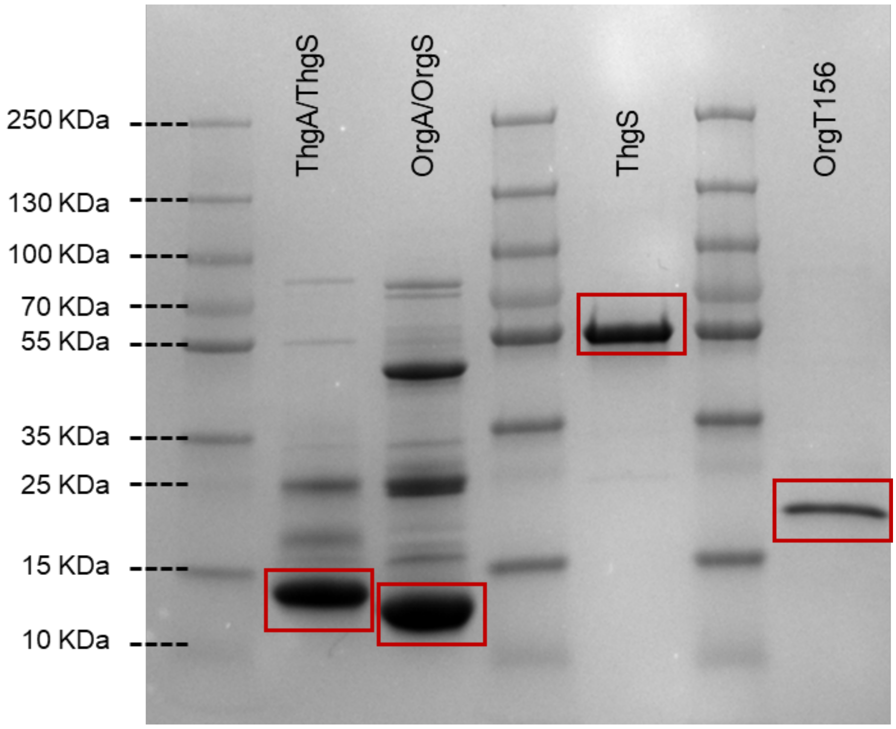

Supplementary Fig. S2. SDS-PAGE gel of purified peptides and proteins.

SDS-PAGE gel showing Ni-NTA elution fractions. 1) Ladder. 2) His_6-ThgA (8.64 kDa) co-expressed with ThgS. 3) His_6-OrgA (8.51 kDa) co-expressed with OrgS. 4) PageRuler™ Prestained Protein Ladder. 5) His_6-ThgS (55.5 kDa) 6) His_6-OrgT156 (21 kDa). Peptides ThgA and OrgA showed a higher apparent mass than expected mass. Masses of both peptides were confirmed using ESI mass spectrometry.

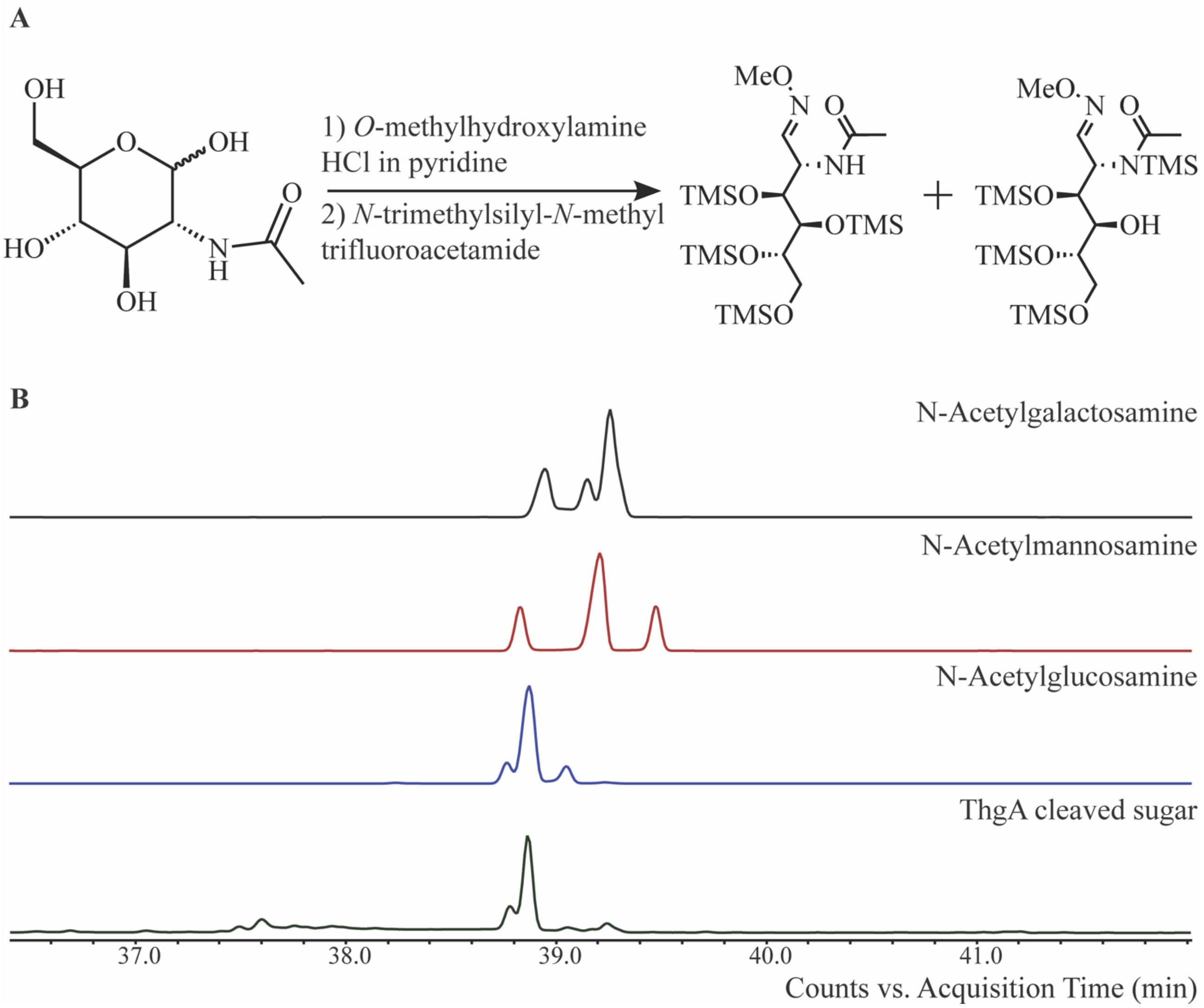

Supplementary Fig. S3. Identification of ThgA glycosylation using GC-MS.

A) *N*-Acetyl-hexosamine derivatization scheme. During step 1 of the reaction, both *E* and *Z* isomers of the methyloxime are formed (only one is drawn). During step 2 of the reaction a mixture of 4 and 5 trimethylsilyl containing derivatives form because the acetyl group will only partially react (Mairinger et al., 2020). MS analysis of thus derivatized sugars typically do not show the molecular ion as the most intense ion, but instead fragment ions such as an ion at m/z 319 (see SI of (Oman et al., 2011)). The three peaks observed at m/z 319 in the EIC result from fragmentation of the two major products (*E* and *Z* with 4 TMS groups) and a minor product with 5 TMS groups. B) GC-MS analysis of the sugar cleaved from ThgA after derivatization as explained in the methods section (bottom) in comparison to N-acetylated hexosamine standards derivatized similarly. The EIC was tracked at both m/z 319 and 274 as a secondary qualifier fragment to minimize background from an interfering compound.

A

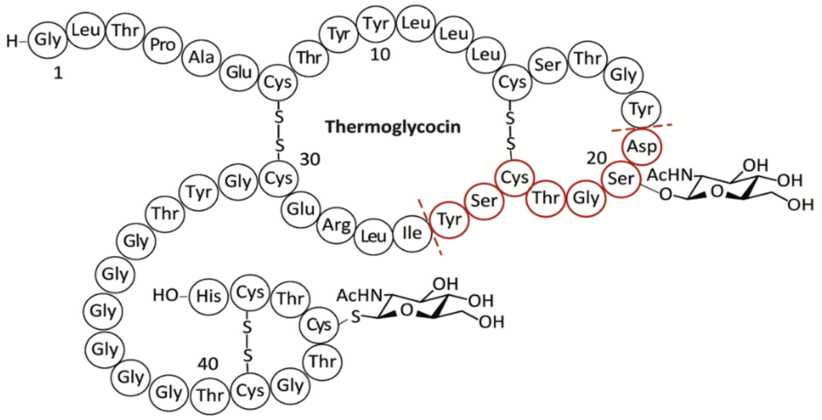

B

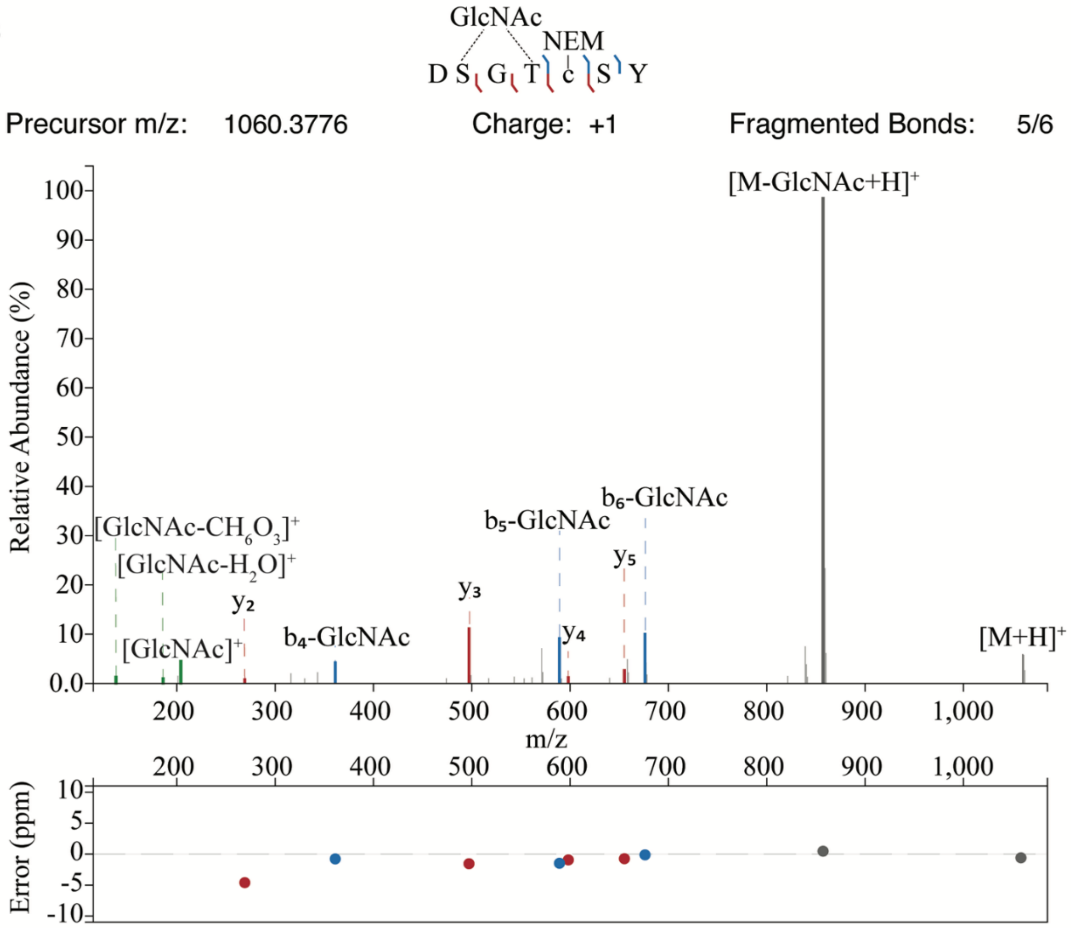

Supplementary Fig. S4. Chymotrypsin digest fragment of mThgA.

(A) Drawing of thermoglycocin to illustrate the proteolytic fragment shown in panel B. (B) ESI-LC-MS/MS spectrum of mThtA fragment encompassing residues 19 to 25 generated by chymotrypsin digest. $(M+H)^+$ calculated monoisotopic *m/z*: 1,060.3776, observed *m/z*: 1,060.3773; $(M - GlcNAc+H)^+$ calculated monoisotopic *m/z*: 857.2982, observed *m/z*: 857.2986. mThtA was alkylated with NEM and digested by chymotrypsin. Fragmentation data shows the GlcNAc is cleaved from the peptide during fragmentation, before the peptide bonds are broken. All b-ion fragments lack GlcNAc. Figure prepared using the Interactive Peptide Annotator Webtool (Brademan et al., 2019). For calculated and observed ions, see Table S4.

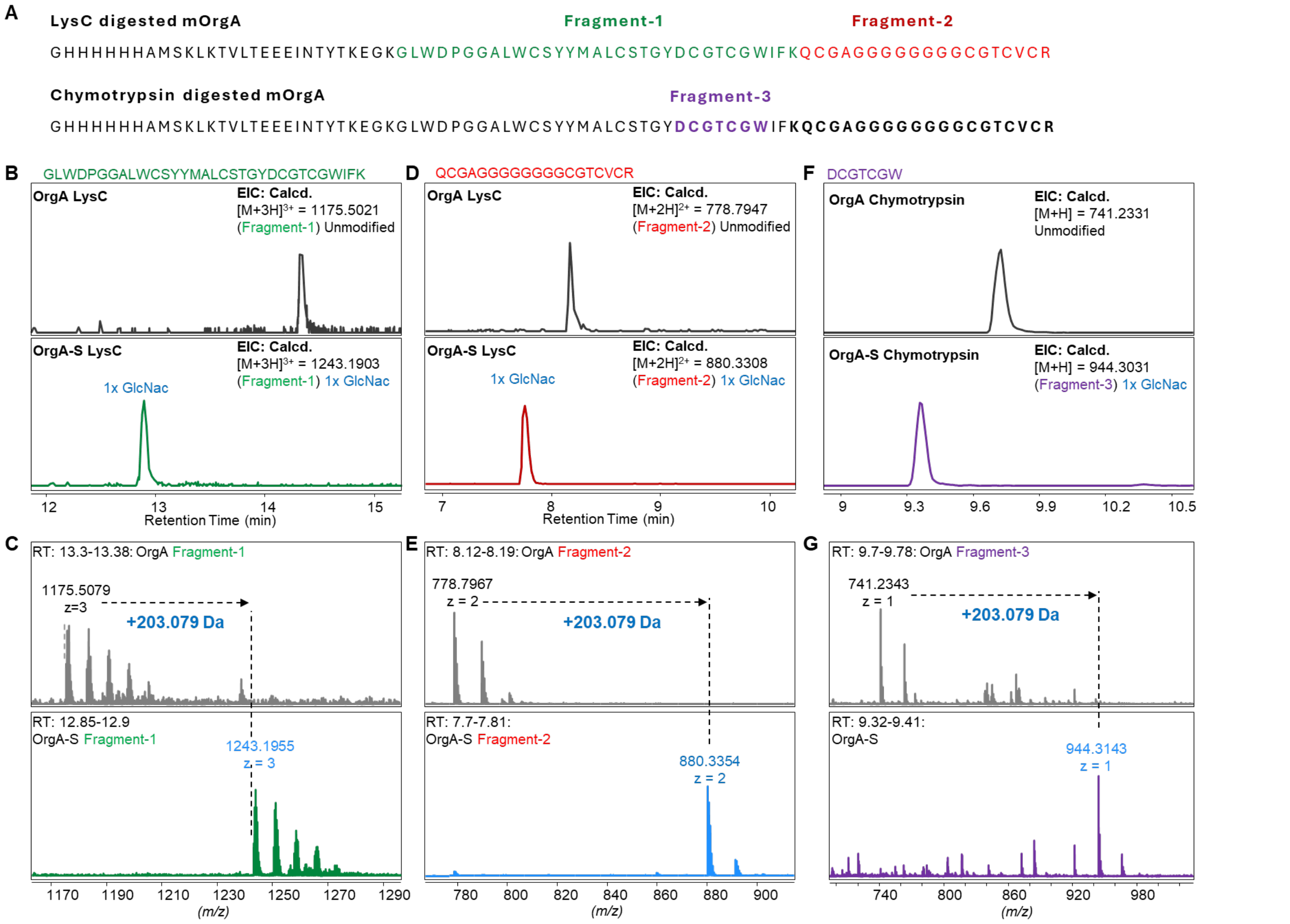

Supplementary Fig. S5. ESI-HR-MS analysis of LysC and chymotrypsin-digested mOrgA in the presence of TCEP.

(A) OrgA sequence highlighting fragments resulting from LysC (fragment-1: green, fragment-2: red font) and chymotrypsin (fragment-3: purple font) digestions. (B) Extracted ion chromatogram (EIC) of fragment-1 from unmodified OrgA (top, grey trace) and mOrgA i.e. OrgA co-expressed with OrgS (bottom, green trace). Calculated monoisotopic $[M+3H]^{3+}$ EIC values are shown; ($[M+H]^{+}$ = 3524.4916 Da for unmodified, 3727.571 Da for 1x glycosylation). The data show that fragment-1 has one glycosylation. (C) HR-MS spectra of fragment-1 from OrgA (top, grey) and mOrgA (bottom, green) peptides showing the observed m/z and the mass shift for 1x GlcNAcylation. (D) EIC of fragment-2 from unmodified OrgA (top, grey trace) and mOrgA (bottom, blue trace). Calculated $[M+2H]^{2+}$ EIC values are shown; ($[M+H]^{+}$ = 1556.5821 Da for unmodified, 1759.6615 Da for 1x glycosylation). The data show that fragment-2 has one glycosylation. (E) HR-MS spectra of fragment-2 from OrgA (top, grey) and mOrgA (bottom, grey) peptides showing the observed m/z and the mass shift for 1x GlcNAcylation. (F) EIC of fragment-3 from unmodified OrgA (top, grey trace) and mOrgA (bottom, purple trace). Calculated $[M+H]^{+}$ EIC values are shown. The data show that fragment-3 has one glycosylation. (G) HR-MS spectra of fragment-3 from OrgA (top, grey) and mOrgA (bottom, purple) peptides showing the observed m/z and the mass shift for 1x GlcNAcylation.

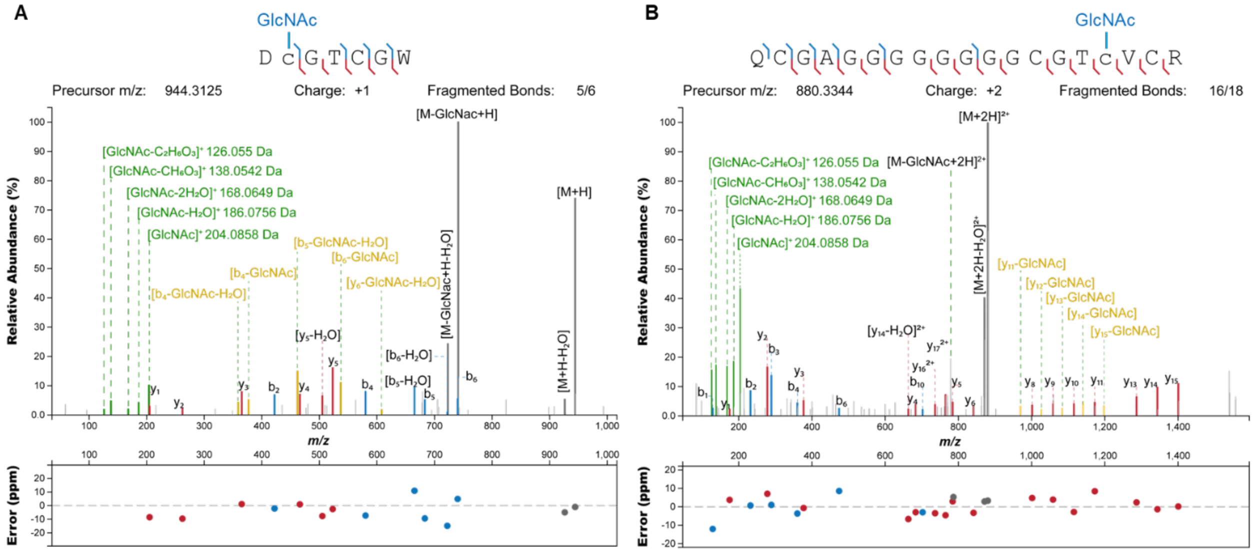

Supplementary Fig. S6. ESI-HR-MS/MS analysis of LysC and chymotrypsin-digested mOrgA in the presence of TCEP.

(A) HR-MS/MS fragmentation pattern of the chymotrypsin-digest fragment-3 (Fig. S5) observed as monoisotopic $[M+H]^+$ = 944.3115 Da (theoretical: $[M+H]^+$ = 944.3125 Da) showing GlcNAcylation at Cys24, and (B) of the LysC-digest fragment-2 (Fig. S5) observed as monoisotopic $[M+2H]^{2+}$ = 880.3373 Da (theoretical: $[M+2H]^{2+}$ = 880.3344 Da); ($[M+H]^+$ = 1759.6615 Da) showing another GlcNAcylation at Cys48 with the representative b-ions in blue and y-ions in red. Neutral loss of GlcNAc was also observed and the corresponding fragments are depicted in yellow. Immonium ions of GlcNAc fragments are depicted in green. For calculated and observed ions, see Tables S5 and S6.

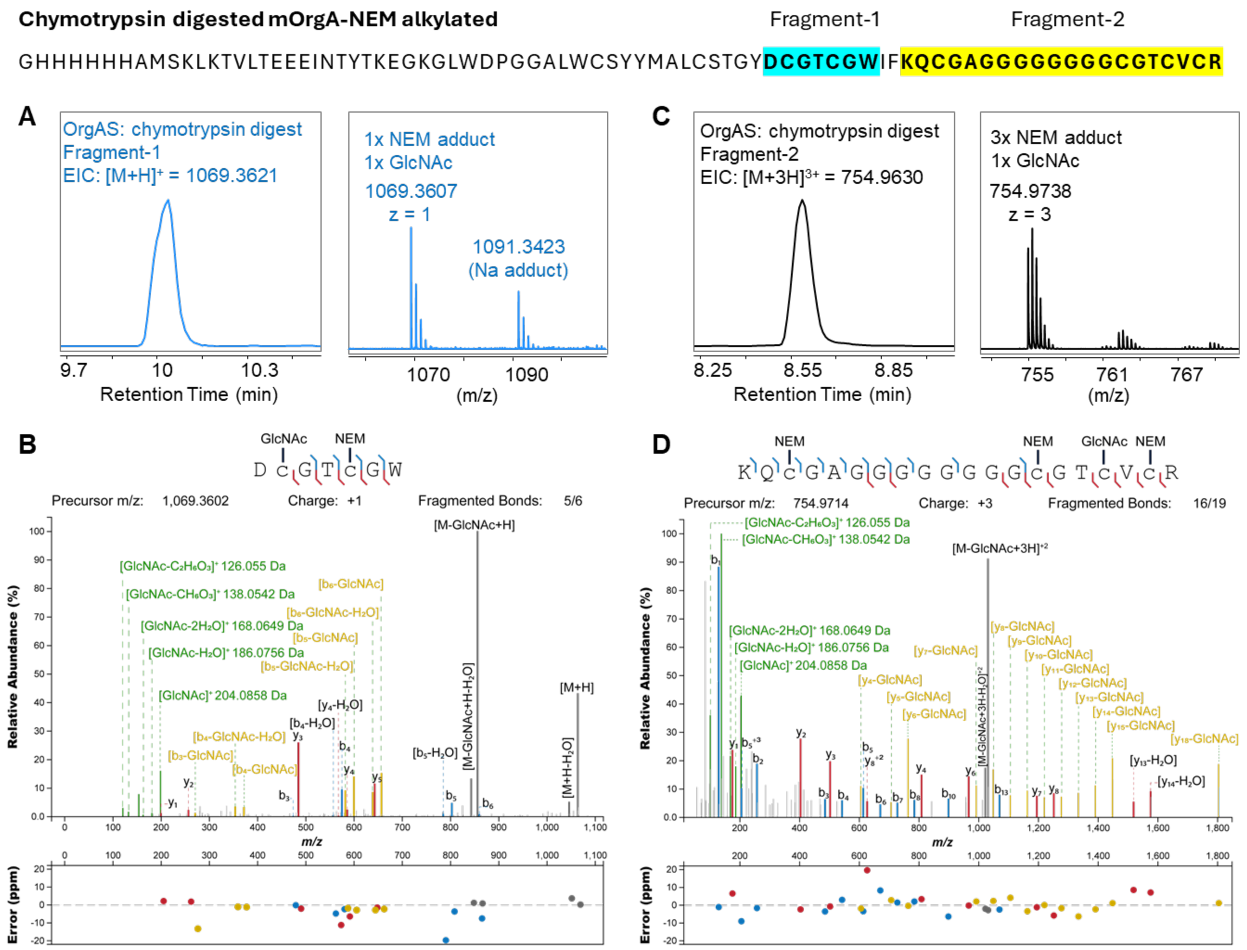

Supplementary Fig. S7. ESI-HR-MS/MS analysis of NEM-alkylated, chymotrypsin-digested mOrgA in presence of TCEP.

(A) Extracted ion chromatogram (EIC) of the chymotrypsin-digest fragment-1 (highlighted in blue) from mOrgA (left) corresponding to the mass of mono GlcNAcylation and 1x NEM-adduct in fragment-1. Calculated monoisotopic $[M+H]^+$ = 1069.3602 Da EIC values are shown in the left trace. HR-MS spectra of fragment-1 showing the observed monoisotopic mass $[M+H]^+$ = 1069.3607 Da is displayed on right. (B) HR-MS/MS fragmentation pattern of fragment-1 showing mono GlcNAcylation at Cys24 and mono NEM-alkylation at Cys27 with the representative b-ions in blue and y-ions in red. Neutral loss of GlcNAc was also observed and the corresponding fragments are depicted in yellow. Immonium ions of GlcNAc fragments are depicted in green. (C) EIC of the chymotrypsin-digest fragment-2 (highlighted in yellow) from mOrgA (left) corresponding to the mass of mono GlcNAcylation and three NEM adducts in fragment-2. Calculated monoisotopic $[M+3H]^{3+}$ = 754.9714 Da EIC values are shown in the left trace; deconvoluted $[M+H]^+$ = 2262.8901 Da. HR-MS spectra of fragment-2 showing the observed monoisotopic mass is displayed on the right. (D) HR-MS/MS fragmentation pattern of fragment-2 showing mono GlcNAcylation at Cys48 and three NEM alkylations on Cys residues at positions 34, 45 and 50, with the representative b-ions in blue and y-ions in red. Neutral loss of GlcNAc was also observed and the corresponding fragments are depicted in yellow. Immonium ions of GlcNAc fragments are depicted in green. For tables of the observed and calculated ions, see Tables S7 and S8.

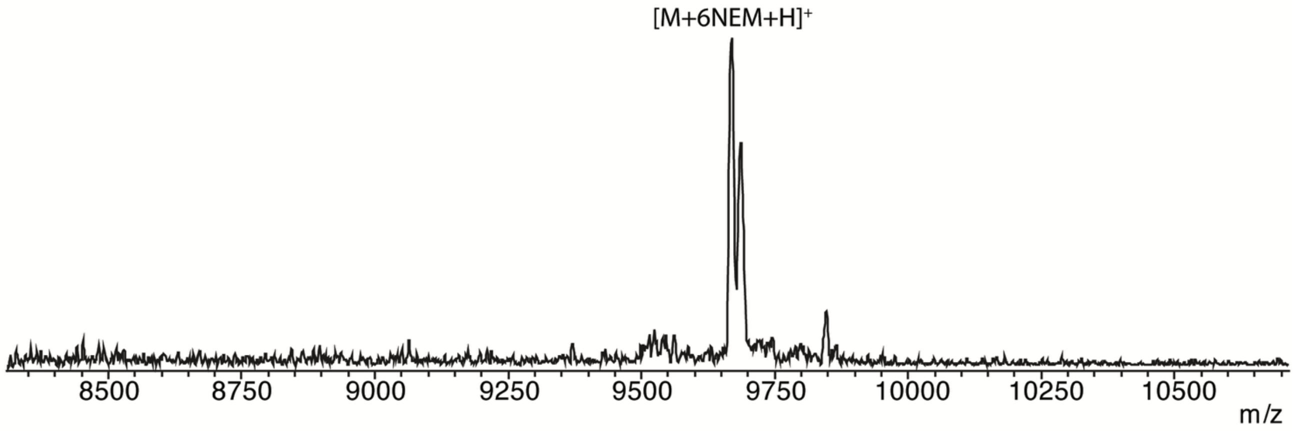

Supplementary Fig. S8. NEM alkylation of mThgA.
ThgA was bisglycosylated via *in vitro* reaction with ThgS and UDP-GlcNAc in the presence of TCEP. Free Cys were then alkylated with NEM. $[M+6NEM+H]^+$ Calculated average mass: 9669.7 Da, Observed mass: 9669.1 Da.

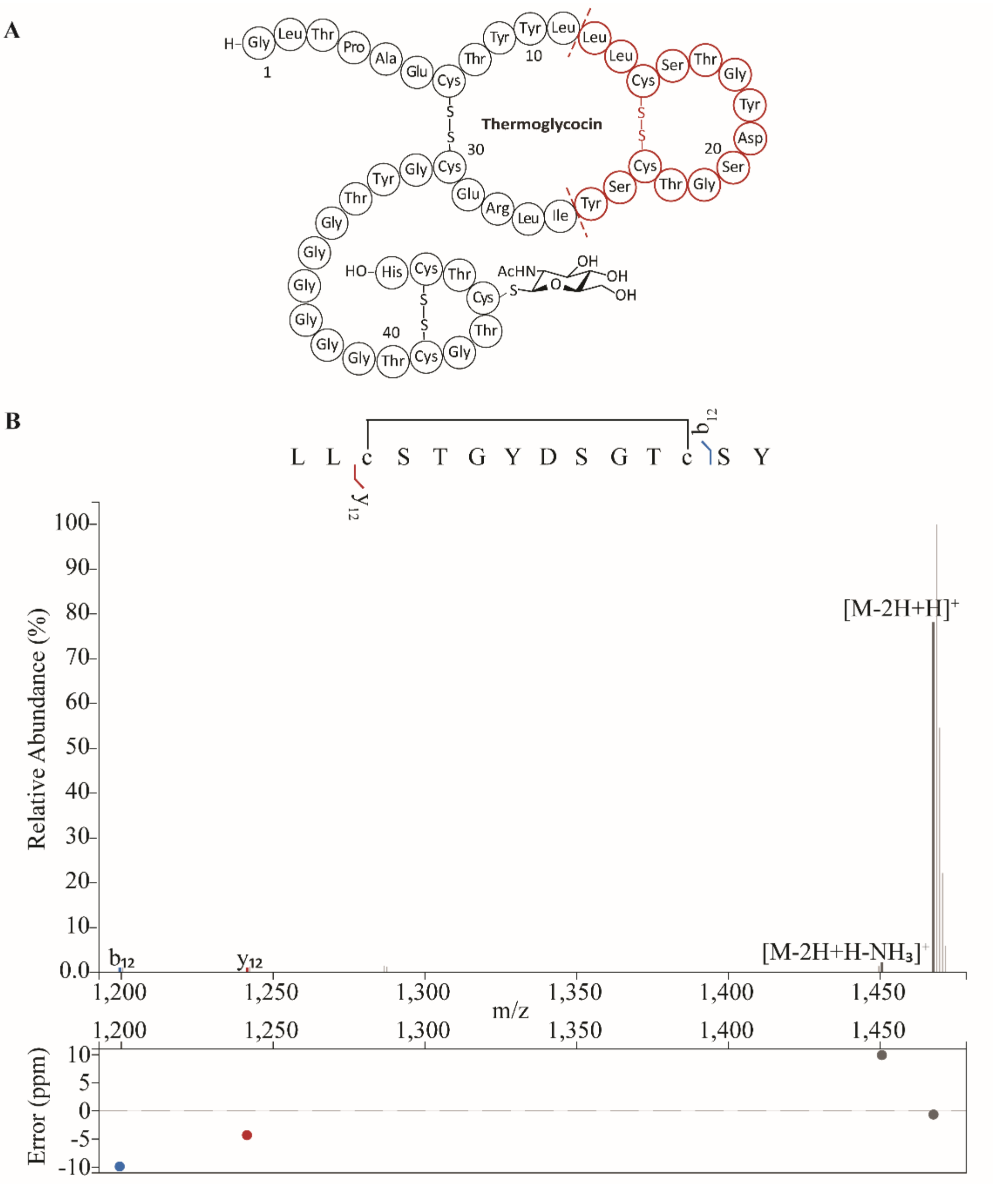

Supplementary Fig. S9. LC MS/MS data suggesting a disulfide bond between Cys14 and Cys23 of ThgA.

(A) Drawing of thermoglycocin to illustrate the fragment shown in panel B. (B) LC-MS/MS of thermolysin-digested monoglycosylated ThgA fragment containing residues 12-25 with a disulfide bond connecting Cys14 and Cys23, $(M\text{-}2H\text{+}H)^+$ drawn in panel A: calculated monoisotopic *m/z*: 1467.5767, observed *m/z*: 1467.5757. Figure prepared using the Interactive Peptide Annotator Webtool (Brademan et al., 2019).

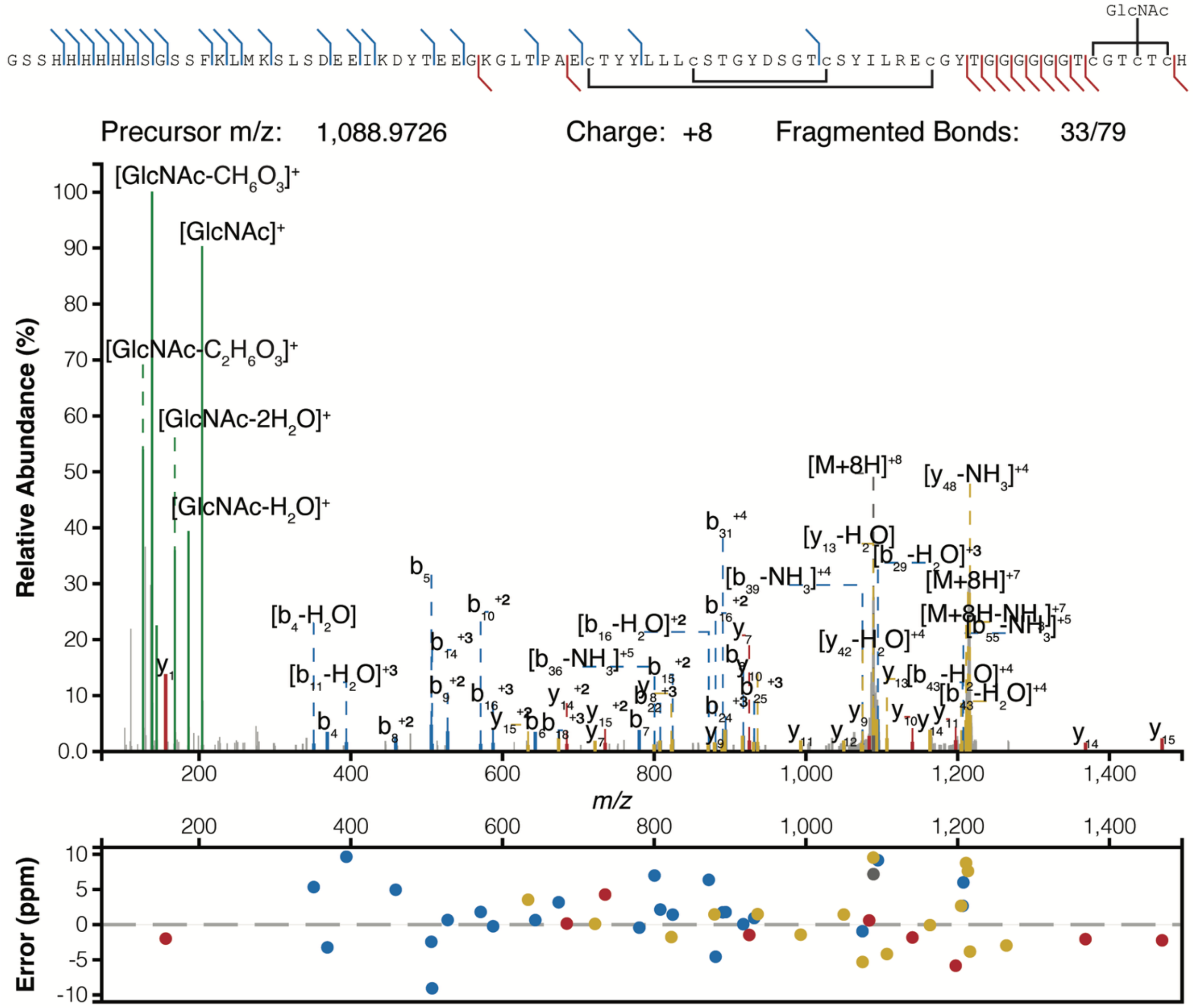

Supplementary Fig. S10. LC-MS/MS of ThgA showing less fragmentation in proposed disulfide bonded areas.

LC-MS/MS data of monoglycosylated ThgA (observed monoisotopic mass: $[M+8H]^{8+}$: 1088.9804, calculated monoisotopic mass $[M+8H]^{8+}$: 1088.9726; calculated monoisotopic mass for $[M+H]^{+}$: 8704.7464; observed deconvoluted mass for $[M+H]^{+}$: 8704.7883) expressed in *E. coli* carrying one GlcNAc modification. Peaks in yellow show b and y ions for which the GlcNAc has been cleaved off during fragmentation. Peaks shown in green are GlcNAc and its fragmentation products. Prepared using the Interactive Peptide Annotator Webtool (Brademan et al., 2019). For calculated and observed ions, see Table S9.

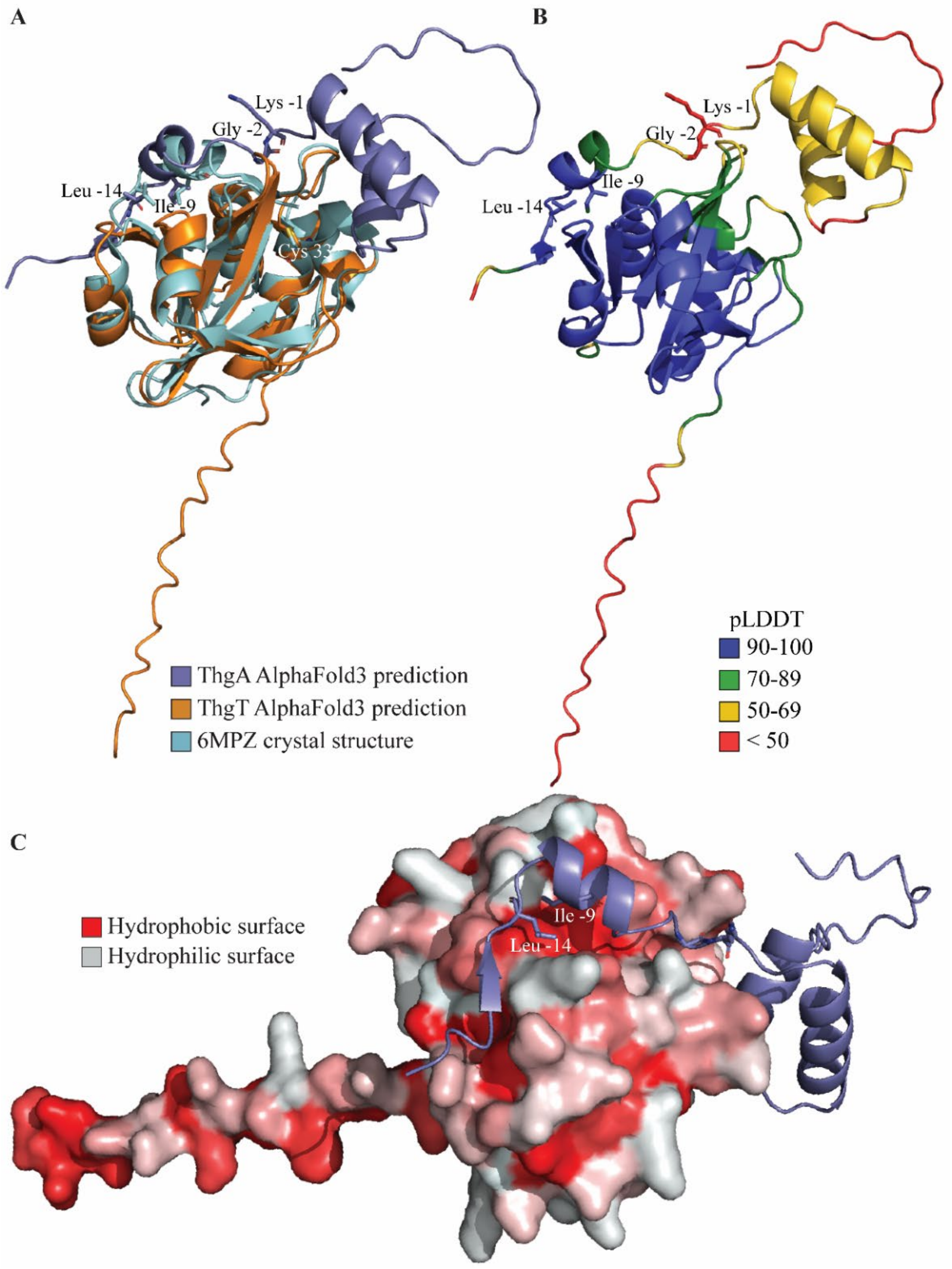

Supplementary Fig. S11. ThgA/ThgT157 predicted structure.

(A) Structural alignment of the LahT150 crystal structure with a covalently bound inhibitor (GLZ) based on the LahA sequence (PDB ID: 6MPZ) with the AlphaFold3 prediction of ThgT157 (residues 1-157) and ThgA leader peptide complex (RMSD= 1.127). Stick diagram showing the hydrophobic residues of the ThgA leader peptide at positions −9 and −14 overlap with the hydrophobic residues of the LahA leader peptide at positions −7 and −12. The GG motif of LahA (shown here as Gly and covalently bound amino-acetaldehyde) binds in a similar fashion as the GK motif in ThgA. (B) AlphaFold3 prediction of ThgA in complex with ThgT157 colored by pLDDT. (C) Surface of AlphaFold3 prediction of ThgT157, colored by hydrophobicity, with ThgA (in cartoon representation) highlighting the location of hydrophobic residues at -9 and -14 that occupy two hydrophobic pockets of ThgT157.

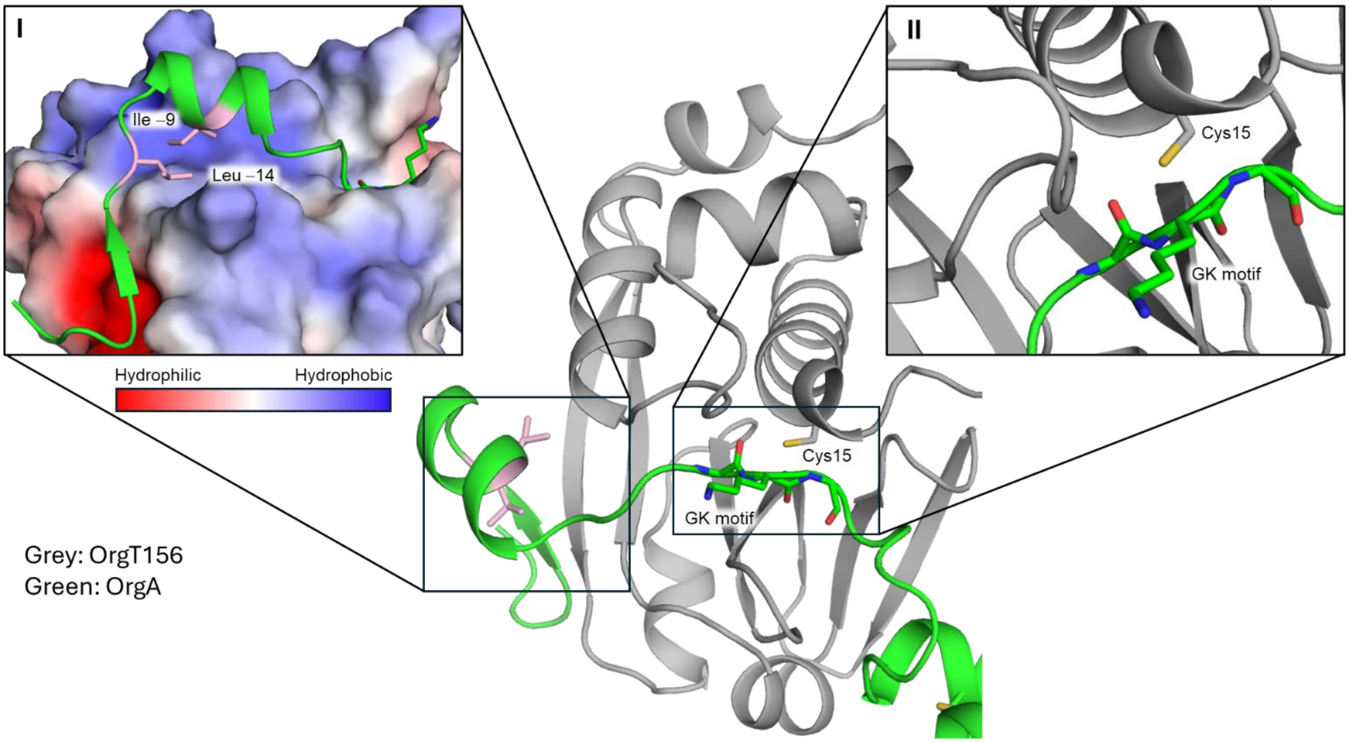

Supplementary Fig. S12. Predicted structure of the OrgT156/OrgA complex.

AlphaFold 3 predicted structure of OrgT156 (grey) in complex with OrgA (green) is shown in the middle as a cartoon representation. (I) The Adaptive Poisson-Boltzmann Solver (APBS) electrostatic map of OrgT156 is shown as a zoomed-in image. The Ile -9 and Leu -14 side chains (pink) in the OrgA leader peptide are shown occupying hydrophobic pockets. (II) A close-up view of the OrgT156 active site displaying the catalytic Cys15 nucleophile in close proximity to the GK motif of OrgA where proteolytic cleavage occurs.

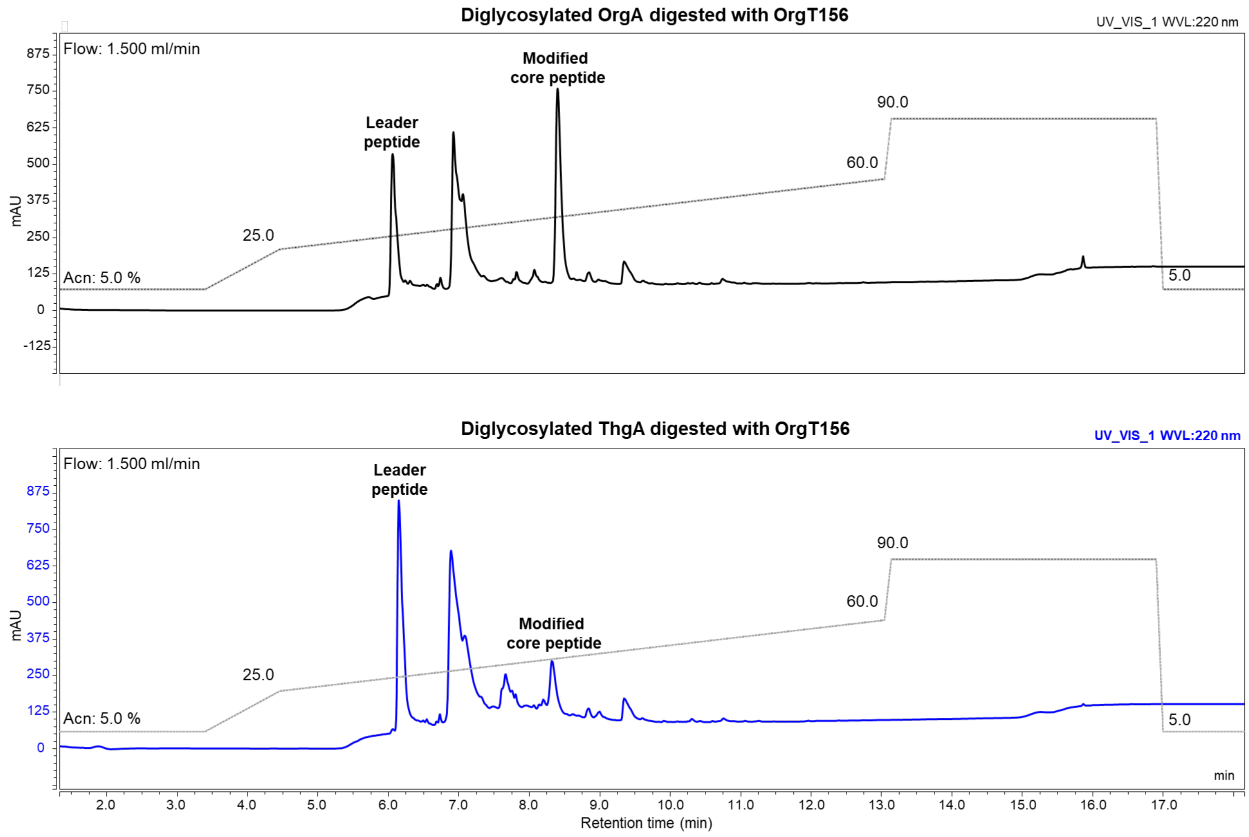

Supplementary Fig. S13. UHPLC trace of fully modified peptides.

(A) UHPLC trace of bisglycosylated OrgA digested by OrgT. (B) UHPLC trace of bisglycosylated ThgA digested by OrgT156. For both assays, an Accucore^{TM} C18 column (150 × 4.6 mm, 2.6 µm particle size) was used.

Table S1. Plasmids and gblocks used in this study

Plasmid Construct	Vector Backbone (Cloning site)	5' to 3' sequence	
His$_6$-ThgA:ThgS	pRSFDuet-1 MCS-1	ORF (ThgA)<br>5' and 3' flanking sequences from plasmid backbone	GGGGAATTGTGAGCGGATAACAATTCCCCTGTAGAAATAATTTTGTTTAACTTTAATAAGGA<br>GATATACC<br>ATGGGCAGCAGCCATCACCATCATCACCACAGCGGCAGCAGCTTTAAGCTGATGAAGTCGCT<br>TTCAGACGAAGAAATCAAAGATTATACCGAAGAAGGTAAGGGGCTTACTCCCGCCGAATGC<br>ACATACTACTTGCTGTTATGCAGCACTGGTTATGATTCTGGAACATGCTCGTACATTCTGCGC<br>GAGTGCGGGTACACAGGTGGCGGAGGGGGTGGAACGTGCGGCACATGTACGTGTCATTAA<br>AGCCAGGATCCGAATTCGAGCTCGGCGCGCCTGCAGGTCGACAAGCTTGCGGCCGCATAATG<br>CTTAAGTC
Translated sequence	MGSSHHHHHHSGSSFKLMKSLSDEEIKDYTEEGKGLTPAECTYYLLLCSTGYDSGTCSYILRECGYTGGGGGGTCGTCTCH*		
	MCS-2	ORF (ThgS)<br>5' and 3' flanking sequences from plasmid backbone	AGGGGAATTGTGAGCGGATAACAATTCCCCATCTTAGTATATTAGTTAAGTATAAGAAGGAG<br>ATATACAT<br>ATGAGCGGCAGCAGCGAAAACCTGTACTTCCAATCCTATAACAAAGGCCTCAACATCTATCT<br>TGAAAAGAACTATCCGTCAGTTAAGAAGTTTACTAGTAAGATTAATTACTTCTACGACCATAT<br>TAACTCGATCACGAATAACATCTCTTACATCTTGGACCAGAACTGCTACTCGGATGAGATGA<br>ACTTTAACCTCAACTCATTCAAGCGCTTCGAGAGCATCAAATCGACGATCTCAGTAGTGGTG<br>ATGTGCAAGAATGAGGAGCGCTGTATCGCACGTTGTATCTCTAGCATTATTAAGACATTGGA<br>CACCAATGACGAGCTGATTGTGCTCGACACAGGGAGTAATGATGATACGCTCAATATTCTGG<br>AGCGCGAATTTCCAAGCGTGGTCATCATCAAGGAGAAGTGGAATAACGACTTTGGGTCTATG<br>CGTAATATTGGGATTGATAAGGCAAAGAATAAATGGGTATTCTTCATCGACGCGGACGAAAT<br>TCTGGACAGCAACAGCATGCGTAGCTTGAAGTTATACCTCAAGGTTATTGACTTTATGGGTCT<br>TGAGAATGTCGTCATCAATCCAATCATCGTCAATAAGAATTCGCACATTGTACAAGGTGTCC<br>GTCGTATCATCAAGAAGTCCGACCGTATTCGTTACTATGGCCTCATCCATGAAGAACCCCGTT<br>TGGACAAGAATATGTATGGCAAGGACGTCGACTCCATCTCTTTTGACAACGTTATCTTGTATC<br>ACGACGGTTATACTAAGAAGGTTATGAACGAGAAGAACAAGTACATCCGCAACACCGAGTT<br>GCTGAAGAAGATGATGATGTTAGAGCCTGAGTATCCCCGCTGGATTTATTTCTACTGCCGTGA<br>CGGTAAGAACCTCATCTCTGAAGAGGATTATGAGAAGTATTTAAACCAGGTCATCAGCCTGT<br>GCCGTGATGATAAATACTATGAGGAGTATAAGATCCGCGCGTTATCAAACCTTATTGAGCAA<br>TACCTTATTAAGGGCAATGTAGATGAAGCCGAGAAGAAGCTTAGTGAGTTAAAGGAGATTTG<br>TAGCGACTTGAGTGACGTGTTTTATTTTGACACTTACATCCAACTCGTTAAGATCAAATATAA<br>CTGCCATCTTTTGTTAAAGAAGGCGATCTCATATCGCCAGAGTCGTAATAACATCGAGTTTGG<br>CAGCATTCATTCGAATTACTTCCACATTGACTACCTCATTGCTAAATTGTTCTTTGAGGTGGGT<br>GAGTACCAAAAGAGTTTCAATATTTTGAAGAAGCTTGAGATGAATGAGTTTCGCGACTATAA<br>AGAGGACTATAAACAACTGTATCACAGCCTTAACAAGTACTTCGATAATGTGAATTTCAAAT<br>AA<br>GCAGATCTCAATTGGATATCGGCCGGCCACGCGATCGCTGACGTCGGTACCCTCGAGTCTGG<br>TAAAGAAAC
Translated sequence	MSGSSENLYFQSYNKGLNIYLEKNYPSVKKFTSKINYFYDHINSITNNISYILDQNCYSDEMNFNLNSFKRFESIKSTISVVVMCKNE<br>ERCIARCISSIIKTLDTNDELIVLDTGSNDDTLNILEREFPSVVIIKEKWNNDFGSMRNIGIDKAKNKWVFFIDADEILDSNSMRSLKL<br>YLKVIDFMGLENVVINPIIVNKNSHIVQGVRRIIKKSDRIRYYGLIHEEPRLDKNMYGKDVDSISFDNVILYHDGYTKKVMNEKNK<br>YIRNTELLKKMMMLEPEYPRWIYFYCRDGKNLISEEDYEKYLNQVISLCRDDKYYEEYKIRALSNLIEQYLIKGNVDEAEKKLSEL<br>KEICSDLSDVFYFDTYIQLVKIKYNCHLLLKKAISYRQSRNNIEFGSIHSNYFHIDYLIAKLFFEVGEYQKSFNILKKLEMNEFRDYK<br>EDYKQLYHSLNKYFDNVNFK*		
His$_6$-ThgS	pRSFDuet-1	ORF (ThgS)<br>5' and 3' flanking sequences from backbone	GGGGAATTGTGAGCGGATAACAATTCCCCTGTAGAAATAATTTTGTTTAACTTTAATAAGGA<br>GATATACC<br>ATGGGCAGCAGCCATCACCATCATCACCACAGCGGCAGCAGCGAAAACCTGTACTTCCAATC<br>CTATAACAAAGGCCTCAACATCTATCTTGAAAAGAACTATCCGTCAGTTAAGAAGTTTACTA<br>GTAAGATTAATTACTTCTACGACCATATTAACTCGATCACGAATAACATCTCTTACATCTTGG<br>ACCAGAACTGCTACTCGGATGAGATGAACTTTAACCTCAACTCATTCAAGCGCTTCGAGAGC<br>ATCAAATCGACGATCTCAGTAGTGGTGATGTGCAAGAATGAGGAGCGCTGTATCGCACGTTG<br>TATCTCTAGCATTATTAAGACATTGGACACCAATGACGAGCTGATTGTGCTCGACACAGGGA<br>GTAATGATGATACGCTCAATATTCTGGAGCGCGAATTTCCAAGCGTGGTCATCATCAAGGAG<br>AAGTGGAATAACGACTTTGGGTCTATGCGTAATATTGGGATTGATAAGGCAAAGAATAAATG<br>GGTATTCTTCATCGACGCGGACGAAATTCTGGACAGCAACAGCATGCGTAGCTTGAAGTTAT<br>ACCTCAAGGTTATTGACTTTATGGGTCTTGAGAATGTCGTCATCAATCCAATCATCGTCAATA<br>AGAATTCGCACATTGTACAAGGTGTCCGTCGTATCATCAAGAAGTCCGACCGTATTCGTTACT<br>ATGGCCTCATCCATGAAGAACCCCGTTTGGACAAGAATATGTATGGCAAGGACGTCGACTCC<br>ATCTCTTTTGACAACGTTATCTTGTATCACGACGGTTATACTAAGAAGGTTATGAACGAGAAG<br>AACAAGTACATCCGCAACACCGAGTTGCTGAAGAAGATGATGATGTTAGAGCCTGAGTATCC<br>CCGCTGGATTTATTTCTACTGCCGTGACGGTAAGAACCTCATCTCTGAAGAGGATTATGAGAA<br>GTATTTAAACCAGGTCATCAGCCTGTGCCGTGATGATAAATACTATGAGGAGTATAAGATCC<br>GCGCGTTATCAAACCTTATTGAGCAATACCTTATTAAGGGCAATGTAGATGAAGCCGAGAAG<br>AAGCTTAGTGAGTTAAAGGAGATTTGTAGCGACTTGAGTGACGTGTTTTATTTTGACACTTAC<br>ATCCAACTCGTTAAGATCAAATATAACTGCCATCTTTTGTTAAAGAAGGCGATCTCATATCGC<br>CAGAGTCGTAATAACATCGAGTTTGGCAGCATTCATTCGAATTACTTCCACATTGACTACCTC<br>ATTGCTAAATTGTTCTTTGAGGTGGGTGAGTACCAAAAGAGTTTCAATATTTTGAAGAAGCTT

			GAGATGAATGAGTTTCGCGACTATAAAGAGGACTATAAACAACTGTATCACAGCCTTAACAA<br>GTACTTCGATAATGTGAATTTCAAATAA<br>AGCCAGGATCCGAATTCGAGCTCGGCGCGCCTGCAGGTCGACAAGCTTGCGGCCGCATAATG<br>CTTAAGTC
Translated sequence	MGSSHHHHHHSGSSENLYFQSYNKGLNIYLEKNYPSVKKFTSKINYFYDHINSITNNISYILDQNCYSDEMNFNLNSFKRFESIKSTI<br>SVVVMCKNEERCIARCISSIIKTLDTNDELIVLDTGSNDDTLNILEREFPSVVIIKEKWNNDFGSMRNIGIDKAKNKWVFFIDADEIL<br>DSNSMRSLKLYLKVIDFMGLENVVINPIIVNKNSHIVQGVRRIIKKSDRIRYYGLIHEEPRLDKNMYGKDVDSISFDNVILYHDGYT<br>KKVMNEKNKYIRNTELLKKMMMLEPEYPRWIYFYCRDGKNLISEEDYEKYLNQVISLCRDDKYYEEYKIRALSNLIEQYLIKGNV<br>DEAEKKLSELKEICSDLSDVFYFDTYIQLVKIKYNCHLLLKKAISYRQSRNNIEFGSIHSNYFHIDYLIAKLFFEVGEYQKSFNILKKL<br>EMNEFRDYKEDYKQLYHSLNKYFDNVNFK*		
His$_6$-ThgA	pRSFDuet-1	ORF (ThgA)<br>5' and 3' flanking sequences from backbone	GGGGAATTGTGAGCGGATAACAATTCCCCTGTAGAAATAATTTTGTTTAACTTTAATAAGGA<br>GATATACC<br>ATGGGCAGCAGCCATCACCATCATCACCACAGCGGCAGCAGCTTTAAGCTGATGAAGTCGCT<br>TTCAGACGAAGAAATCAAAGATTATACCGAAGAAGGTAAGGGGCTTACTCCCGCCGAATGC<br>ACATACTACTTGCTGTTATGCAGCACTGGTTATGATTCTGGAACATGCTCGTACATTCTGCGC<br>GAGTGCGGGTACACAGGTGGCGGAGGGGGTGGAACGTGCGGCACATGTACGTGTCATTAA<br>TAAAGCCAGGATCCGAATTCGAGCTCGGCGCGCCTGCAGGTCGACAAGCTTGCGGCCGCATA<br>ATGCTTAA
Translated sequence	MGSSHHHHHHSGSSFKLMKSLSDEEIKDYTEEGKGLTPAECTYYLLLCSTGYDSGTCSYILRECGYTGGGGGGTCGTCTCH*		
MBP-ThgT 33-157	pACYCDuet-1	ORF<br>5' and 3' flanking sequences from backbone	GGGGAATTGTGAGCGGATAACAATTCCCCTGTAGAAATAATTTTGTTTAACTTTAATAAGGA<br>GATATACCATG<br>AAAATCGAAGAAGGTAAACTGGTAATCTGGATTAACGGCGATAAAGGCTATAACGGTCTCGC<br>TGAAGTCGGTAAGAAATTCGAGAAAGATACCGGAATTAAAGTCACCGTTGAGCATCCGGATA<br>AACTGGAAGAGAAATTCCCACAGGTTGCGGCAACTGGCGATGGCCCTGACATTATCTTCTGG<br>GCACACGACCGCTTTGGTGGCTACGCTCAATCTGGCCTGTTGGCTGAAATCACCCCGGACAA<br>AGCGTTCCAGGACAAGCTGTATCCGTTTACCTGGGATGCCGTACGTTACAACGGCAAGCTGA<br>TTGCTTACCCGATCGCTGTTGAAGCGTTATCGCTGATTTATAACAAAGATCTGCTGCCGAACC<br>CGCCAAAAACCTGGGAAGAGATCCCGGCGCTGGATAAAGAACTGAAAGCGAAAGGTAAGAG<br>CGCGCTGATGTTCAACCTGCAAGAACCGTACTTCACCTGGCCGCTGATTGCTGCTGACGGGG<br>GTTATGCGTTCAAGTATGAAAACGGCAAGTACGACATTAAAGACGTGGGCGTGGATAACGCT<br>GGCGCGAAAGCGGGTCTGACCTTCCTGGTTGACCTGATTAAAAACAAACACATGAATGCAGA<br>CACCGATTACTCCATCGCAGAAGCTGCCTTTAATAAAGGCGAAACAGCGATGACCATCAACG<br>GCCCGTGGGCATGGTCCAACATCGACACCAGCAAAGTGAATTATGGTGTAACGGTACTGCCG<br>ACCTTCAAGGGTCAACCATCCAAACCGTTCGTTGGCGTGCTGAGCGCAGGTATTAACGCCGC<br>CAGTCCGAACAAAGAGCTGGCGAAAGAGTTCCTCGAAAACTATCTGCTGACTGATGAAGGTC<br>TGGAAGCGGTTAATAAAGACAAACCGCTGGGTGCCGTAGCGCTGAAGTCTTACGAGGAAGA<br>GTTGGCGAAAGATCCACGTATTGCCGCCACCATGGAAAACGCCCAGAAAGGTGAAATCATGC<br>CGAACATCCCGCAGATGTCCGCTTTCTGGTATGCCGTGCGTACTGCGGTGATCAACGCCGCCA<br>GCGGTCGTCAGACTGTCGATGAAGCCCTGAAAGACGCGCAGACTAATTCGAGCTCCCACCAT<br>CACCATCACCACGCGAATTCGGTACCGCTGGTTCCGCGTGGATCTGAGAACCTGTACTTCCAA<br>TCCGATTGCGGCCCGGCATGCGTGAGCATGTTGCTGGATTATATGTTCGATCTGAAATTCACC<br>ATTGGCGAACTGAAGAATGTGGTATACAGTAACGCGAATGGTTGCACCTTCCTGGGCATTAA<br>ACGTGGTCTTGAGAAACTGGGTATCAAATCTACTGTCTATAAATGCGAGAATGACATCAGTA<br>TTTTCAATGAGGCGATTTATCCTTTTCTGACGCAGATTTCTACGGACTCGGTGAAACACTTTA<br>TTGTTGTGTACGGCCGCAAGAAGAATAAACTGATCATCTGTGACCCGTCCAAGAACGGAATT<br>GAATACTGGAAGTTAAACAACCTGATGAAAATTTGGCAGCCCTATATCCTGCGTATCAGCAA<br>GGAGATCTAA<br>TCGAACAGAAAGTAATCGTATTGTACACGGCCGCATAATCGAAATTAATACGACTCACTATA<br>GGGGAATT
Translated sequence	MKIEEGKLVIWINGDKGYNGLAEVGKKFEKDTGIKVTVEHPDKLEEKFPQVAATGDGPDIIFWAHDRFGGYAQSGLLAEITPDKA<br>FQDKLYPFTWDAVRYNGKLIAYPIAVEALSLIYNKDLLPNPPKTWEEIPALDKELKAKGKSALMFNLQEPYFTWPLIAADGGYAF<br>KYENGKYDIKDVGVDNAGAKAGLTFLVDLIKNKHMNADTDYSIAEAAFNKGETAMTINGPWAWSNIDTSKVNYGVTVLPTFKG<br>QPSKPFVGVLSAGINAASPNKELAKEFLENYLLTDEGLEAVNKDKPLGAVALKSYEEELAKDPRIAATMENAQKGEIMPNIPQMS<br>AFWYAVRTAVINAASGRQTVDEALKDAQTNSSSHHHHHHANSVPLVPRGSENLYFQSDCGPACVSMLLDYMFDLKFTIGELKNV<br>VYSNANGCTFLGIKRGLEKLGIKSTVYKCENDISIFNEAIYPFLTQISTDSVKHFIVVYGRKKNKLIICDPSKNGIEYWKLNNLMKIW<br>QPYILRISKEI*		

OrgA	pRSFDuet (MCS-1)	ORF<br>5’ and 3’ flanking sequences from backbone	GGAATTGTGAGCGGATAACAATTCCCCATCTTAGTATATTAGTTAAGTATAAGAAGGAGATA<br>TACATATG<br>ATGAGCGGCAGCAGCGAAAACCTGTACTTCCAATCCTATAACAAAGGCCTCAACATCTATCT<br>TGAAAAGAACTATCCGTCAGTTAAGAAGTTTACTAGTAAGATTAATTACTTCTACGACCATAT<br>TAACTCGATCACGAATAACATCTCTTACATCTTGGACCAGAACTGCTACTCGGATGAGATGA<br>ACTTTAACCTCAACTCATTCAAGCGCTTCGAGAGCATCAAATCGACGATCTCAGTAGTGGTG<br>ATGTGCAAGAATGAGGAGCGCTGTATCGCACGTTGTATCTCTAGCATTATTAAGACATTGGA<br>CACCAATGACGAGCTGATTGTGCTCGACACAGGGAGTAATGATGATACGCTCAATATTCTGG<br>AGCGCGAATTTCCAAGCGTGGTCATCATCAAGGAGAAGTGGAATAACGACTTTGGGTCTATG<br>CGTAATATTGGGATTGATAAGGCAAAGAATAAATGGGTATTCTTCATCGACGCGGACGAAAT<br>TCTGGACAGCAACAGCATGCGTAGCTTGAAGTTATACCTCAAGGTTATTGACTTTATGGGTCT<br>TGAGAATGTCGTCATCAATCCAATCATCGTCAATAAGAATTCGCACATTGTACAAGGTGTCC<br>GTCGTATCATCAAGAAGTCCGACCGTATTCGTTACTATGGCCTCATCCATGAAGAACCCCGTT<br>TGGACAAGAATATGTATGGCAAGGACGTCGACTCCATCTCTTTTGACAACGTTATCTTGTATC<br>ACGACGGTTATACTAAGAAGGTTATGAACGAGAAGAACAAGTACATCCGCAACACCGAGTT<br>GCTGAAGAAGATGATGATGTTAGAGCCTGAGTATCCCCGCTGGATTTATTTCTACTGCCGTGA<br>CGGTAAGAACCTCATCTCTGAAGAGGATTATGAGAAGTATTTAAACCAGGTCATCAGCCTGT<br>GCCGTGATGATAAATACTATGAGGAGTATAAGATCCGCGCGTTATCAAACCTTATTGAGCAA<br>TACCTTATTAAGGGCAATGTAGATGAAGCCGAGAAGAAGCTTAGTGAGTTAAAGGAGATTTG<br>TAGCGACTTGAGTGACGTGTTTTATTTTGACACTTACATCCAACTCGTTAAGATCAAATATAA<br>CTGCCATCTTTTGTTAAAGAAGGCGATCTCATATCGCCAGAGTCGTAATAACATCGAGTTTGG<br>CAGCATTCATTCGAATTACTTCCACATTGACTACCTCATTGCTAAATTGTTCTTTGAGGTGGGT<br>GAGTACCAAAAGAGTTTCAATATTTTGAAGAAGCTTGAGATGAATGAGTTTCGCGACTATAA<br>AGAGGACTATAAACAACTGTATCACAGCCTTAACAAGTACTTCGATAATGTGAATTTCAAAT<br>AA<br>GCAGATCTCAATTGGATATCGGCCGGCCACGCGATCGCTGACGTCGGTACCCTCGAGTCTGG<br>TAAAGAAA
Translated sequence	MHHHHHHAMSKLKTVLTEEEINTYTKEGKGLWDPGGALWCSYYMALCSTGYDCGTCGWIFKQCGAGGGGGGGCGTCVCR*		
OrgA:OrgS	pRSFDuet (MCS-1)	ORF<br>6xHis-OrgA<br>5’ and 3’ flanking sequences from backbone	TAATACGACTCACTATAGGGGAATTGTGAGCGGATAACAATTCCCCTGTAGAAATAATTTTG<br>TTTAACTTTAATAAGGAGATATACC<br>ATGCACCACCACCACCACCACGCAATGTCGAAGCTTAAGACTGTGCTGACAGAAGAGGAGAT<br>TAACACTTATACCAAAGAGGGAAAGGGATTATGGGACCCAGGTGGTGCCTTATGGTGCTCTT<br>ACTACATGGCGCTTTGTAGCACCGGATATGACTGCGGGACTTGCGGTTGGATATTTAAGCAG<br>TGCGGGGCCGGTGGTGGTGGAGGAGGTGGTGGGTGTGGTACATGCGTGTGTCGTTGA<br>AAGCTTGCGGCCGCATAATGCTTAAGTCGAACAGAAAGTAATCGTATTGTACACGGCCGCAT<br>AATCGAAAT
Translated sequence	MHHHHHHAMSKLKTVLTEEEINTYTKEGKGLWDPGGALWCSYYMALCSTGYDCGTCGWIFKQCGAGGGGGGGCGTCVCR*		
	(MCS-2)	ORF<br>6xHis-OrgA<br>5’ and 3’ Flanking sequences from backbone	TAATACGACTCACTATAGGGGAATTGTGAGCGGATAACAATTCCCCTGTAGAAATAATTTTG<br>TTTAACTTTAATAAGGAGATATACC<br>ATGCACCACCACCACCACCACGCAATGTCGAAGCTTAAGACTGTGCTGACAGAAGAGGAGAT<br>TAACACTTATACCAAAGAGGGAAAGGGATTATGGGACCCAGGTGGTGCCTTATGGTGCTCTT<br>ACTACATGGCGCTTTGTAGCACCGGATATGACTGCGGGACTTGCGGTTGGATATTTAAGCAG<br>TGCGGGGCCGGTGGTGGTGGAGGAGGTGGTGGGTGTGGTACATGCGTGTGTCGTTGA<br>AAGCTTGCGGCCGCATAATGCTTAAGTCGAACAGAAAGTAATCGTATTGTACACGGCCGCAT<br>AATCGAAAT
Translated sequence	MKGMPKNTKTAKKFFEHLKLYSPNISENIRHLELNSFDNLLLKEPSLSVIIMCKDEQRCIARCLNAIKNNIGINDEVIVIDTGSADDT<br>LNILNNFSMTNKYSIIETSWNNDFAEIRNLGISQATKDWIVFIDADETIEKGSFENLKSHLSIVDTLNTTVVCCPAIVNSGGHVVQTV<br>RRIIPNNDSVFYFGMVHEEPRHINKSELNFLAFDDVILHHDGYMKSVSSAKQKQERNTSLLYTMIDKEPLNPKWYYLLCRDGKGV<br>LEENFYRDSLLKVITLCGQAPFYEEYKLRALSDLIAHYLSAGDIENAKYYLEDLKKLAPNMTDTLYWDIFIQLIIFEYAYHQFIQQII<br>DYKQTNKGLDYGSLNSNGFHLDYLLSQLFFNIRDYNSCFNILKKLEDAQYGEYQENYMELYDALQKYLLQIGEENEKKIFHKTT*		
OrgT156	pET28a(+) (NdeI-XhoI)	ORF<br>OrgS<br>5’ and 3’ flanking sequences from backbone	TAATACGACTCACTATAGGGGAATTGTGAGCGGATAACAATTCCCCATCTTAGTATATTAGTT<br>AAGTATAAGAAGGAGATATACAT<br>ATGAAAGGCATGCCGAAAAACACTAAAACCGCAAAAAAATTCTTCGAACATCTGAAACTGT<br>ACTCGCCAAATATTAGCGAAAATATCCGCCACCTGGAACTGAACAGCTTTGACAATCTGCTG<br>CTGAAAGAACCGAGCTTGTCCGTTATTATCATGTGTAAAGATGAACAGCGTTGTATTGCCCGT<br>TGCTTGAACGCAATCAAAAATAACATTGGCATTAACGATGAGGTCATCGTTATTGACACCGG<br>CAGCGCGGATGATACTCTGAACATCCTGAACAACTTCTCCATGACCAATAAATATTCTATTAT<br>TGAAACTAGCTGGAATAACGATTTTGCCGAAATCCGTAATTTAGGCATTAGCCAGGCCACTA<br>AAGATTGGATTGTCTTTATTGACGCTGACGAAACCATTGAAAAGGGATCATTCGAAAATCTG<br>AAGTCCCACCTGTCCATCGTGGACACCTTAAATACCACGGTAGTGTGTTGTCCGGCAATCGTA<br>AATTCGGGAGGGCATGTGGTGCAGACGGTTCGCCGCATTATCCCAAACAACGATAGCGTGTT<br>CTATTTTGGGATGGTGCATGAGGAGCCGCGCCATATCAATAAATCGGAATTGAACTTTCTTGC<br>TTTCGATGATGTGATTCTCCATCACGACGGGTATATGAAAAGTGTCTCTTCTGCCAAACAAAA<br>ACAGGAACGCAATACGTCTCTGCTTTACACCATGATTGATAAAGAACCGCTGAACCCGAAAT<br>GGTACTATTTATTATGCCGTGATGGAAAAGGTGTGCTGGAGGAAAATTTTTACCGTGATTCAT<br>TGCTTAAAGTGATTACCCTGTGTGGCCAGGCCCCATTTTATGAAGAATACAAACTGCGTGCGC<br>TGAGCGATCTGATCGCTCACTATCTTAGCGCCGGTGATATTGAGAACGCGAAATACTACCTG<br>GAAGATTTAAAAAAATTAGCTCCGAACATGACGGATACGCTGTATTGGGACATCTTCATTCA<br>ACTGATCATTTTCGAATATGCTTATCACCAATTTATTCAGCAAATTATTGATTATAAACAGAC<br>CAACAAAGGACTTGATTACGGTAGCTTAAACTCGAACGGCTTCCACTTAGATTATCTGCTGA<br>GCCAGCTGTTTTTAACATCCGCGATTATAACTCGTGTTTTAACATTTTGAAAAAACTGGAGG

			ATGCCCAGTACGGCGAATATCAAGAGAACTATATGGAACTTTATGATGCCCTGCAGAAATAT CTTTTGCAGATTGGCGAGGAAAACGAAAAAAAAATTTTTCACAAAACGACCTAA CTCGAGTCTGGTAAAGAAACCGCTGCTGCGAAATTTGAACGCCAGCACATGGACTCGTCTAC TAGCGCAGCTTAATTAACCTAGGCTGCTGCCACCGCTGAGCAATAA
Translated sequence	MGSSHHHHHHSSGLVPRGSHMEGLYFQSMKKKYFTKQLEENDCGPAAVSMLIKYSWDIEMTLSQLKVILSTNKNGTTFLGIKRG LQKLGVETNVSKCVSSKETFQELKYPCITQIKGSNNHFITLFKATKNHVYIGDPSKNQIKRMKINRFLSNWVPFVLEVEKLIDSQKL TSYSITETKQSNT*		

Table S2. Fragment ions observed in Fig. 3B.

Theoretical mass of GlcNAcylated peptide with two NEM adducts $[M+2H]^{2+}$: 861.3137. Color codes: y-ions in red, b-ions in blue, ions that underwent the loss of the GlcNAc in dark yellow and GlcNAc fragments in green.

Ion	Number	Modifications; Mass Change	Neutral Loss	Charge	Intensity	Exp'tal m/z	Theor. m/z	Mass Error (ppm)
b	1		-H2O	1	4073.33	84.0443	84.0444	-1.3
y	1			1	15051.7	156.0765	156.0768	-1.4
b	2			1	8176.53	159.0766	159.0764	0.9
b	3		-H2O	1	2219.37	198.0870	198.0873	-1.6
b	3			1	6139.88	216.0978	216.0979	-0.2
b	4		-H2O	1	2500.68	255.1095	255.1088	2.9
b	4			1	6948.52	273.1197	273.1193	1.2
b	5		-H2O	1	7536.14	312.1303	312.1302	0.0
b	5			1	4791.73	330.1409	330.1408	0.3
b	6		-H2O	1	9783.93	369.1519	369.1517	0.4
y	2	<NEM; 125.0477>		1	5457.92	384.1336	384.1336	-0.1
b	6			1	2940.06	387.1622	387.1623	-0.3
b	7		-H2O	1	6170.03	426.1732	426.1732	0.1
b	7			1	1794.9	444.1832	444.1837	-1.1
y	3	<NEM; 125.0477>		1	4413.12	485.1815	485.1813	0.5
b	8		-H2O	1	5876.67	527.2208	527.2209	-0.1
b	8			1	2851.84	545.2311	545.2314	-0.6
y-GlcNAc	4	<NEM; 125.0477>	-GlcNAc	1	2574.99	588.1906	588.1905	0.2
y	4	<hexNAc; 203.0794><br><NEM; 125.0477>		1	3480	791.2694	791.2699	-0.6
y-GlcNAc	5	<NEM; 125.0477>	-GlcNAc	1	926.42	689.2377	689.2382	-0.7
y	5	<hexNAc; 203.0794><br><NEM; 125.0477>		1	1254	892.3167	892.3176	-1.0
y-GlcNAc	6	<NEM; 125.0477>	-GlcNAc	1	2885.16	746.2605	746.2597	1.2
y	6	<hexNAc; 203.0794><br><NEM; 125.0477>		1	4314	949.3390	949.3391	0.0
b	9	<NEM; 125.0477>	-H2O	1	2481.16	755.2764	755.2777	-1.8
M+2H-GlcNAc				2	19169.1	759.7746	759.7740	0.8
b	9	<NEM; 125.0477>		1	1819.84	773.2845	773.2883	-5.0
b	10	<NEM; 125.0477>	-H2O	1	1152.53	812.2965	812.2992	-3.3
M+2H				2	30087.62	861.3147	861.3137	1.2
b	11	<NEM; 125.0477>	-H2O	1	1140.9	913.3463	913.3469	-0.7
b	11	<NEM; 125.0477>		1	949.78	931.3540	931.3575	-3.7
y-GlcNAc	7	<NEM; 125.0477><br><NEM; 125.0477>	-GlcNAc	1	2907	974.3155	974.3165	-1.0
y	7	<NEM; 125.0477><br><hexNAc; 203.0794><br><NEM; 125.0477>		1	4215	1177.3954	1177.3959	-0.5
y-GlcNAc	8	<NEM; 125.0477><br><NEM; 125.0477>	-GlcNAc	1	1161	1075.3633	1075.3642	-0.9
y	8	<NEM; 125.0477><br><hexNAc; 203.0794><br><NEM; 125.0477>		1	1935	1278.4406	1278.4436	-2.4
y-GlcNAc	9	<NEM; 125.0477><br><NEM; 125.0477>	-GlcNAc	1	1494	1132.3852	1132.3857	-0.5
y	9	<NEM; 125.0477><br><hexNAc; 203.0794>		1	2275	1335.4651	1335.4651	0.0

		<NEM; 125.0477>						
y-GlcNAc	10	<NEM; 125.0477><br><NEM; 125.0477>	-GlcNAc	1	1501	1189.4065	1189.4072	-0.6
y	10	<NEM; 125.0477><br><hexNAc; 203.0794><br><NEM; 125.0477>		1	2111	1392.4842	1392.4866	-1.7
y	11	<NEM; 125.0477><br><hexNAc; 203.0794><br><NEM; 125.0477>		1	1214	1449.5066	1449.5080	-1.0
y-GlcNAc	11	<NEM; 125.0477><br><NEM; 125.0477>	-GlcNAc	1	1513	1246.4275	1246.4286	-0.9
y-GlcNAc	12	<NEM; 125.0477><br><NEM; 125.0477>	-GlcNAc	1	1498	1303.4485	1303.4501	-1.2
y-GlcNAc	13	<NEM; 125.0477><br><NEM; 125.0477>	-GlcNAc	1	2452	1360.4697	1360.4715	-1.3
b-GlcNAc	14	<NEM; 125.0477><br><NEM; 125.0477>	-GlcNAc	1	958	1363.4708	1363.4712	-0.3
y-GlcNAc	14	<NEM; 125.0477><br><NEM; 125.0477>	-GlcNAc	1	915	1417.4920	1417.4930	-0.7
M+2H-GlcNAc			-NH3	1	940.2	1502.5280	1502.5214	4.4
M+2H-GlcNAc				1	17867.66	1519.5427	1519.5480	-3.4
HexNAc				1	82831.61	204.0869	204.0872	-1.5
HexNAc-H2O				1	32585.21	186.0760	186.0766	-3.2
HexNAc-2H2O				1	28166.63	168.0656	168.0661	-3.1
HexNAc-C6H10O3N				1	9226.08	144.0656	144.0661	-3.1
HexNAc- C7H8O2N				1	46640.46	138.0550	138.0555	-3.8
HexNAc-C6H8O2N				1	30084.02	126.0550	126.0555	-3.6

Table S3. Fragments observed in Figure 3C (observed monoisotopic $[M+3H]^{3+}$ = 1159.5014 Da; theoretical mass 1159.4925 Da). Deconvoluted monoisotopic [M] = 3,475.4775 which accounts for the LTPAECTYYL fragment (monoisotopic [M] = 1172.5424 containing 1x disulfide (-2.01 Da) that connects via a disulfide to the ILRECGYTGGGGGGTCGTCTCH fragment (monoisotopic [M] = 2101.8438 Da modified with 1x GlcNAc (203.0794 Da). Color codes: y-ions in red, b-ions in blue and z•-ions in yellow originating from the LTPAECTYYL fragment and ions from the ILRECGYTGGGGGGTCGTCTCH fragment in dark yellow.

Sequence		Theoretical Mz	Charge					
LTPAECTYYL		1159.4925	3					
**Ion**	**Nr.**	**Modification**	**Neutral Loss**	**Charge**	**Intensity**	**Experimental Mz**	**Theoretical Mz**	**Mass Error (ppm)**
a	2		-H2O	1	426.22	169.1326	169.1335	-5.541
a	2			1	4324.05	187.1436	187.1441	-2.6816
b	2			1	5932.62	215.1385	215.139	-2.4006
b	3		-H2O	1	137.46	294.1815	294.1812	0.9661
b	4			1	211.91	383.2283	383.2289	-1.5512
b	5			1	259.65	512.2713	512.2715	-0.3601
b	9	1x disulfide; ILRECGYTGGGGGGTCGTCTCH +GlcNAc		3	102.26	1115.8048	1115.7943	9.4499
M+3H			-H2O	3	431.73	1153.5054	1153.4889	14.2676
M+3H				3	2924.76	1159.5014	1159.4925	7.7068
y	8	1x disulfide; ILRECGYTGGGGGGTCGTCTCH +GlcNAc		3	4247.47	1088.1248	1088.1152	8.8066
y	9	1x disulfide; ILRECGYTGGGGGGTCGTCTCH +GlcNAc		3	218.75	1121.8102	1121.7978	11.0739
y	5	1x disulfide; ILRECGYTGGGGGGTCGTCTCH +GlcNAc		2	154.67	1483.1094	1483.103	4.3464
y	6	1x disulfide; ILRECGYTGGGGGGTCGTCTCH +GlcNAc		2	114.68	1547.6252	1547.6242	0.6146
y	7	1x disulfide; ILRECGYTGGGGGGTCGTCTCH +GlcNAc		2	147.56	1583.1526	1583.1428	6.1865
y	8	1x disulfide; ILRECGYTGGGGGGTCGTCTCH +GlcNAc		2	981.45	1631.6828	1631.6692	8.3425
y	21	1x disulfide; +GlcNAc + LTPAECTYYL		3	218.75	1121.8102	1121.7978	11.0578
y	18	1x disulfide; +GlcNAc + LTPAECTYYL		2	154.67	1483.1094	1483.0792	20.38
y	19	1x disulfide; +GlcNAc + LTPAECTYYL		2	114.68	1547.6252	1547.6005	15.9797
z	8	1x disulfide; ILRECGYTGGGGGGTCGTCTCH +GlcNAc		3	343.94	1082.7902	1082.7756	13.4445

Table S4. Fragment ions observed in Fig S4. Color codes: y-ions in red, b-ions in blue, ions that underwent the loss of the GlcNAc in dark yellow.

Ion	Number	Modifications; Mass Change	Neutral Loss	Charge	Intensity	Experimental Mz	Theoretical Mz	Mass Error (ppm)
M+H				1	3939.11	1060.3773	1060.3776	-0.3071
M+H			- HexNAc	1	65281	857.2986	857.2982	0.4746
b	6	<NEM;125.0477>	- HexNAc	1	6799.94	676.2242	676.2243	-0.116
y	5	<NEM;125.0477>		1	1914.01	655.2387	655.2392	-0.7389
y	4	<NEM;125.0477>		1	982.4	598.2172	598.2178	-0.9364
b	5	<NEM;125.0477>	- HexNAc	1	6223.17	589.1914	589.1923	-1.4757
y	3	<NEM;125.0477>		1	7512.33	497.1693	497.1701	-1.5491
b	4		- HexNAc	1	2962.63	361.1351	361.1354	-0.7739
y	2			1	690.13	269.112	269.1132	-4.6083

Table S5. HR-MS/MS table for chymotrypsin-digested mOrgA fragment-3 in presence of TCEP in support of Figure S6A. Color codes: y-ions in red, b-ions in blue, ions that underwent the loss of the GlcNAc in dark yellow.

**Sequence**	**Theoretical Mz**	**Neutral Loss**	**Charge**					
DCGTCGW	944.3125	None	1					
	741.2331	-GlcNAc	1					
**ion**	**Number**	**Modification**	**Neutral Loss**	**Charge**	**Intensity %**	**Experimental Mz**	**Theoretical Mz**	**Error (ppm)**
y	1			1	84.1	205.0954	205.0972	-8.5382
y	2			1	68.16	262.1161	262.1186	-9.5955
b	4		-H2O; -GlcNAc	1	116.32	359.0957	359.102	-17.5042
y	3			1	219.86	365.1282	365.1278	1.0814
b	4		-GlcNAc	1	140.5	377.1114	377.1126	-3.0507
b	2	+GlcNAc		1	192.31	422.1219	422.1228	-2.1474
b	5		-H2O; -GlcNAc	1	404	462.111	462.1112	-0.3804
y	4			1	196.3	466.1759	466.1755	0.8899
b	5		-GlcNAc	1	94.8	480.1126	480.1217	-19.0378
y	5		-H2O	1	179.88	505.1825	505.1864	-7.6892
y	5			1	440.8	523.1956	523.1969	-2.5787
b	6		-GlcNAc	1	300.07	537.1421	537.1432	-2.0562
b	4	+GlcNAc		1	222.1	580.1877	580.192	-7.326
y	6		-H2O	1	48.05	608.1902	608.1956	-8.8367
b	5	+GlcNAc	-H2O	1	264.73	665.1978	665.1906	10.8604
b	5	+GlcNAc		1	145.01	683.1947	683.2011	-9.4269
b	6	+GlcNAc	-H2O	1	29.07	722.2013	722.212	-14.8707
M+H			-H2O; -GlcNAc	1	658.58	723.2237	723.2225	1.6392
b	6	+GlcNAc		1	154.56	740.2262	740.2226	4.8574
M+H			-GlcNAc	1	2709.75	741.2322	741.2331	-1.1861
M+H		+GlcNAc	-H2O	1	149.16	926.2973	926.3019	-4.9816
M+H		+GlcNAc		1	2010.15	944.3115	944.3125	-1.0369

Table S6. HR-MS/MS table for LysC-digested mOrgA fragment-2 in presence of TCEP in support of Figure S6B. Color codes: y-ions in red, b-ions in blue, ions that underwent the loss of the GlcNAc in dark yellow.

**Sequence**				**Theoretical Mz**	**Charge**			
QCGAGGGGGGGGCGTCVCR				880.3344	2			
					%			
**Ion**	**Number**	**Modification**	**Neutral Loss**	**Charge**	**Intensity %**	**Experimental Mz**	**Theoretical Mz**	**Error (ppm)**
b	1			1	136.77	129.0643	129.0659	-12.0595
y	1			1	122	175.1196	175.119	3.7052
b	2			1	439.62	232.0752	232.075	0.6616
y	2			1	841.11	278.1301	278.1281	7.043
b	3			1	695.88	289.0968	289.0965	1.0015
b	4			1	228.73	360.1323	360.1336	-3.6777
y	3			1	267	377.1963	377.1966	-0.6764
b	6			1	135.95	474.1806	474.1766	8.5359
y	14	+GlcNAc	-H2O	2	122.04	663.2508	663.2552	-6.6633
y	4	+GlcNAc		1	205.22	683.2831	683.2851	-2.9931
b	10			1	113.89	702.2603	702.2624	-3.0024
y	16	+GlcNAc		2	199.28	736.2872	736.2898	-3.5187
y	17	+GlcNAc		2	369.98	764.797	764.8005	-4.6062
M+2H				2	978.03	778.799	778.7947	5.5113
y	5	+GlcNAc		1	241.78	784.3351	784.3328	2.9004
y	6	+GlcNAc		1	180.56	841.3515	841.3543	-3.3151
M+2H			-H2O	2	2029.01	871.3316	871.3291	2.84
M+2H				2	5020.6	880.3373	880.3344	3.2853
y	11		-GlcNAc	1	168.08	969.3742	969.3699	4.3996
y	8	+GlcNAc		1	189.64	1001.3897	1001.3849	4.7503
y	12		-GlcNAc	1	111.26	1026.3832	1026.3914	-7.9883
y	9	+GlcNAc		1	208.71	1058.4105	1058.4064	3.867
y	13		-GlcNAc	1	134.1	1083.4059	1083.4129	-6.4271
y	10	+GlcNAc		1	213.36	1115.4247	1115.4279	-2.843
y	14		-GlcNAc	1	239.03	1140.4298	1140.4343	-3.9697
y	11	+GlcNAc		1	236.94	1172.4592	1172.4493	8.4139
y	15		-GlcNAc	1	174.2	1197.4664	1197.4558	8.8595
y	13	+GlcNAc		1	331.84	1286.4953	1286.4923	2.3606
y	14	+GlcNAc		1	498.72	1343.5119	1343.5137	-1.36
y	15	+GlcNAc		1	560.56	1400.5354	1400.5352	0.1491

Table S7. HR-MS/MS table for chymotrypsin-digested NEM-alkylated mOrgA fragment-3 in presence of TCEP in support of Figure S7B. Color codes: y-ions in red, b-ions in blue, ions that underwent the loss of the GlcNAc in dark yellow.

**Sequence**	**Theoretical Mz**	**Charge**						
DCGTCGW	1069.3602	1						
**Ion**	**Number**	**Modifications**	**Neutral Loss**	**Charge**	**Intensity %**	**Experimental Mz**	**Theoretical Mz**	**Error (ppm)**
y	1			1	97	205.0976	205.0972	2.1885
b	3		-H2O; -GlcNAc	1	72.79	258.056	258.0543	6.5654
y	2			1	186.16	262.1191	262.1186	1.8497
b	3		-GlcNAc	1	88.67	276.0612	276.0649	-13.2957
b	4		-H2O; -GlcNAc	1	263.5	359.1016	359.102	-1.0743
b	4		-GlcNAc	1	250	377.1121	377.1126	-1.1945
b	3	+ GlcNAc		1	20.28	479.1442	479.1443	-0.1471
y	3	+NEM		1	2046.77	490.1745	490.1755	-2.0506
b	4	+ GlcNAc	-H2O	1	36.61	562.1787	562.1814	-4.7774
y	4	+NEM	-H2O	1	25.52	573.2062	573.2126	-11.2008
b	4	+ GlcNAc		1	750.58	580.1906	580.192	-2.3276
b	5	+NEM	-H2O; -GlcNAc	1	711	587.1578	587.1589	-1.8322
y	4	+NEM		1	194.54	591.2194	591.2232	-6.4022
b	5	+NEM	-GlcNAc	1	1092.07	605.1677	605.1694	-2.876
b	6	+NEM	-H2O; -GlcNAc	1	664.58	644.1785	644.1803	-2.856
y	5	+NEM		1	915.01	648.2437	648.2446	-1.4642
b	6	+NEM	-GlcNAc	1	1189.94	662.1894	662.1909	-2.272
b	5	+ GlcNAc; NEM	-H2O	1	77.45	790.2227	790.2383	-19.7102
b	5	+ GlcNAc; NEM		1	378.43	808.2459	808.2488	-3.6381
M-GlcNAc-H2O+H			-H2O; -GlcNAc	1	1033.48	848.2712	848.2702	1.1618
b	6	+ GlcNAc; NEM		1	67.07	865.2638	865.2703	-7.5173
M-GlcNAc+H			-GlcNAc	1	7848.18	866.2815	866.2808	0.8321
M+H			-H2O	1	409.98	1051.354	1051.35	3.6958
M+H				1	3400.02	1069.36	1069.36	0.2065

Table S8. HR-MS/MS table for chymotrypsin-digested NEM-alkylated mOrgA fragment-3 in presence of TCEP in support of Figure S7D. Color codes: y-ions in red, b-ions in blue, ions that underwent the loss of the GlcNAc in dark yellow.

Sequence			Theoretical Mz		Charge			
KQCGAGGGGGGGGCGTCVCR			754.9714		3			
**Ion**	**Number**	**Modifications**	**Neutral Loss**	**Charge**	**Intensity %**	**Experimental Mz**	**Theoretical Mz**	**Error (ppm)**
b	1			1	5624.9	129.1021	129.1022	-1.057
y	1			1	1515.52	175.1201	175.119	6.5604
b	5	+NEM		3	660.4	205.0951	205.0969	-8.9996
b	2			1	1206.9	257.1604	257.1608	-1.6195
y	2	+NEM		1	1767.2	403.1749	403.1758	-2.3343
y	13	+2x NEM	-H2O, -GlcNAc	3	385.32	439.1782	439.1708	16.9627
b	3	+NEM		1	413.11	485.216	485.2177	-3.5169
y	3	+NEM		1	1260.77	502.2439	502.2443	-0.7071
b	4	+NEM		1	387.51	542.2408	542.2392	3.0052
y	4	+NEM	-GlcNAc	1	704.11	605.2523	605.2534	-1.892
b	5	+NEM		1	661.82	613.2742	613.2763	-3.3989
y	8	+2x NEM; +GlcNAc		2	361.43	626.2561	626.2438	19.6252
b	6	+NEM		1	295.79	670.3033	670.2977	8.2822
y	5	+NEM	-GlcNAc	1	338.1	706.3031	706.3011	2.796
b	7	+NEM		1	332.25	727.3203	727.3192	1.4953
y	6	+NEM	-GlcNAc	1	1761.09	763.3223	763.3226	-0.3788
b	8	+NEM		1	394.08	784.3423	784.3407	2.0699
y	4	+GlcNAc; +NEM		1	960.06	808.3356	808.3328	3.4081
b	10	+NEM		1	424.53	898.3779	898.3836	-6.3497
y	19	+3x NEM	-H2O, -GlcNAc	2	455.61	957.3583	957.361	-2.7946
y	6	+GlcNAc; +NEM		1	920.25	966.4018	966.402	-0.1957
y	7	+2x NEM	-GlcNAc	1	712.78	991.3815	991.3795	2.0384
M-GlcNAc-H2O+3H			-H2O	2	1103	1021.91	1021.9121	-2.0488
M-GlcNAc+3H				2	5796.8	1030.9145	1030.9174	-2.7898
y	8	+2x NEM	-GlcNAc	1	1073.27	1048.4034	1048.4009	2.3434
b	13	+NEM		1	511.42	1069.4454	1069.448	-2.4279
y	9	+2x NEM	-GlcNAc	1	493.51	1105.427	1105.4224	4.1548
y	10	+2x NEM	-GlcNAc	1	595.06	1162.4399	1162.4439	-3.4162
y	7	+2x NEM; +GlcNAc		1	486.27	1194.4575	1194.4589	-1.1546
y	11	+2x NEM	-GlcNAc	1	449.01	1219.4654	1219.4653	0.0532
y	8	+2x NEM; +GlcNAc		1	542.29	1251.4731	1251.4803	-5.7877
y	12	+2x NEM	-GlcNAc	1	460.43	1276.4846	1276.4868	-1.7228
y	13	+2x NEM	-GlcNAc	1	545.74	1333.4998	1333.5083	-6.3465
y	14	+2x NEM	-GlcNAc	1	716.01	1390.5265	1390.5297	-2.3208
y	15	+2x NEM	-GlcNAc	1	1323.79	1447.5528	1447.5512	1.1114
y	13	+2x NEM; +GlcNAc	-H2O	1	354.36	1518.5901	1518.5771	8.5617
y	16	+2x NEM	-GlcNAc	1	354.36	1518.5901	1518.5883	1.1819
y	14	+2x NEM; +GlcNAc	-H2O	1	585.97	1575.6098	1575.5986	7.1322
y	17	+2x NEM	-GlcNAc	1	585.97	1575.6098	1575.6098	0.0196
y	18	+3x NEM	-GlcNAc	1	1193.02	1803.6687	1803.6667	1.1315

Table S9. MS/MS fragments shown in Figure S10. Fragmentation of GlcNAcylated ThgA with three disulfide bonds: GSSHHHHHHSGSSFKLMKSLSDEEIKDYTEEGKGLTPAECTYYLLLCSTGYDSGT CSYILRECGYTGGGGGGTCGTCTCH theoretical mass $[M+8H]^{8+}$: 1088.9726. Color codes: y-ions in red, b-ions in blue, ions that underwent the loss of the GlcNAc in dark yellow and GlcNAc fragments in green.

ion	Fragment	Modifications; Mass Change	Neutral Loss	Charge	Intensity	Exp'tal m/z	Theoretical m/z	Mass Error (ppm)
y	1			1	20226.73	156.0764	156.0768	-1.9936
b	4		-H2O	1	2096.42	351.1430	351.1411	5.3432
b	4			1	5093.25	369.1505	369.1517	-3.2465
b	11		-H2O	3	2450.36	394.1717	394.1679	9.6577
b	8			2	3883.7	459.1996	459.1973	4.9598
b	5			1	7029.34	506.2094	506.2106	-2.4465
b	14			3	1984.8	507.2110	507.2156	-9.0707
b	9			2	5300.63	527.7271	527.7268	0.6491
b	10			2	1951.93	571.2438	571.2428	1.7996
b	16			3	2267.91	587.6085	587.6086	-0.2339
y-GlcNAc	15	<Cys-Cys;-1.0078><br><Cys-Cys;-1.0078>	-GlcNAc	2	1683	633.7207	633.7185	3.538
b	6			1	5098.98	643.2699	643.2695	0.6398
b	18			3	3496.66	673.9892	673.9871	3.1789
y	14	<Cys-Cys;-1.0078><br><hexNAc; 203.0794><br><Cys-Cys;-1.0078>		2	1952.6	684.7344	684.7343	0.149
y-GlcNAc	7	<Cys-Cys;-1.0078><br><Cys-Cys;-1.0078>	-GlcNAc	1	2784	722.2056	722.2055	0.104
y	15	<Cys-Cys;-1.0078><br><hexNAc;203.0794><br><Cys-Cys;-1.0078>		2	2289.4	735.2613	735.2582	4.274
b	7			1	5636.28	780.3281	780.3284	-0.4337
b	36		-NH3	5	1845.16	800.3818	800.3763	6.9837
b	22			3	2869.59	808.0472	808.0454	2.1408
y-GlcNAc	8	<Cys-Cys;-1.0078><br><Cys-Cys;-1.0078>	-GlcNAc	1	1837.3	823.2517	823.2532	-1.78
b	15			2	3182.43	824.3684	824.3672	1.4217
b	16		-H2O	2	1904.54	871.9095	871.9040	6.3704
y-GlcNAc	9	<Cys-Cys;-1.0078><br><Cys-Cys;-1.0078>	-GlcNAc	1	2413	880.2759	880.2746	1.455
b	16			2	2102.94	880.9052	880.9093	-4.5725
b	31			4	2380.77	890.4093	890.4077	1.7473
b	24			3	5661.44	894.0754	894.0738	1.7745
b	8			1	4124.44	917.3874	917.3873	0.0562
y	7	<Cys-Cys;-1.0078><br><hexNAc;203.0794><br><Cys-Cys;-1.0078>		1	2858.8	925.2835	925.2849	-1.475
b	25			3	2513.56	931.7694	931.7685	0.9336
y-GlcNAc	10	<Cys-Cys;-1.0078><br><Cys-Cys;-1.0078>	-GlcNAc	1	2514.3	937.2975	937.2961	1.49
y-GlcNAc	11	<Cys-Cys;-1.0078><br><Cys-Cys;-1.0078>	-GlcNAc	1	2907.8	994.3161	994.3176	-1.435
y-GlcNAc	12	<Cys-Cys;-1.0078><br><Cys-Cys;-1.0078>	-GlcNAc	1	2838.5	1051.3405	1051.3390	1.435
b	39		-NH3	4	2199.41	1074.5006	1074.5016	-0.9408

y-GlcNAc	42	<Cys-Cys ;-1.0078><br><Cys-Cys ;-1.0078><br><Cys-Cys ;-1.0078><br><Cys-Cys ;-1.0078><br><Cys-Cys ;-1.0078><br><Cys-Cys ;-1.0078>	-H2O<br>-GlcNAc	4	1717.3	1076.1693	1076.1750	-5.32
y	9	<Cys-Cys ;-1.0078><br><hexNAc ;203.0794><br><Cys-Cys ;-1.0078>		1	4089.3	1083.3547	1083.3540	0.592
M+8H				8	4150.79	1088.9804	1088.9726	7.1776
y-GlcNAc	13	<Cys-Cys ;-1.0078><br><Cys-Cys ;-1.0078>	-H2O<br>-GlcNAc	1	11049	1090.3603	1090.3499	9.538
b	29		-H2O	3	8398.26	1094.8527	1094.8426	9.1655
y	10	<Cys-Cys ;-1.0078><br><hexNAc ;203.0794><br><Cys-Cys ;-1.0078>		1	2549.7	1140.3734	1140.3755	-1.835
y	11	<Cys-Cys ;-1.0078><br><hexNAc ;203.0794><br><Cys-Cys ;-1.0078>		1	2913.6	1197.3900	1197.3970	-5.852
b	43	<Cys-Cys ;-1.0078>	-H2O	4	1993.18	1206.5528	1206.5495	2.6743
y-GlcNAc	13	<Cys-Cys ;-1.0078><br><Cys-Cys ;-1.0078>		1	3461.3	1108.3558	1108.3605	-4.191
y-GlcNAc	14	<Cys-Cys ;-1.0078><br><Cys-Cys ;-1.0078>		1	5663.2	1165.3819	1165.3820	-0.076
b-GlcNAc	43	<Cys-Cys ;-1.0078>	-H2O	4	1993.18	1206.5528	1206.5495	2.6743
b	55	<Cys-Cys ;-1.0078><br><Cys-Cys ;-1.0078>	-NH3	5	2836.7	1207.5544	1207.5471	6.006
M+8H-GlcNAc			-NH3	7	8171.34	1213.1070	1213.0964	8.7673
M+8H-GlcNAc				7	9715.37	1215.5380	1215.5287	7.6192
y-GlcNAc	48	<Cys-Cys ;-1.0078><br><Cys-Cys ;-1.0078><br><Cys-Cys ;-1.0078><br><Cys-Cys ;-1.0078><br><Cys-Cys ;-1.0078><br><Cys-Cys ;-1.0078>	-NH3<br>-GlcNAc	4	9487.5	1218.2508	1218.2555	-3.842
y-GlcNAc	15	<Cys-Cys ;-1.0078><br><Cys-Cys ;-1.0078>	-GlcNAc	1	2889	1266.4259	1266.4296	-2.976
y	14	<Cys-Cys ;-1.0078><br><hexNAc ;203.0794><br><Cys-Cys ;-1.0078>		1	2330.5	1368.4585	1368.4614	-2.067
y	15	<Cys-Cys ;-1.0078><br><hexNAc ;203.0794><br><Cys-Cys ;-1.0078>		1	3433.8	1469.5058	1469.5090	-2.238
HexNAc				1	132080.37	204.0863	204.0872	-4.3609
HexNAc-H2O				1	57520.76	186.0756	186.0766	-5.3741
HexNAc-2H2O				1	52529.11	168.0655	168.0661	-3.5105
HexNAc- C6H10O3N				1	32853.98	144.0652	144.0661	-5.9695
HexNAc-C7H8O2N				1	146310.68	138.0548	138.0555	-5.3602
HexNAc- C6H8O2N				1	78811.99	126.0547	126.0555	-6.5051

References

Brademan, D. R., Riley, N. M., Kwiecien, N. W.& Coon, J. J. (2019). Interactive peptide spectral annotator: A versatile web-based tool for proteomic applications. *Mol. Cell. Proteom.* 18, S193.

Consortium, T. U. (2024). UniProt: the universal protein knowledgebase in 2025. *Nucleic Acids Res.* 53, D609.

Main, P., Hata, T., Loo, T. S., Man, P., Novak, P., Havlicek, V., Norris, G. E.& Patchett, M. L. (2020). Bacteriocin ASM1 is an O/S-diglycosylated, plasmid-encoded homologue of glycocin F. *FEBS Lett.* 594, 1196.

Mairinger, T., Weiner, M., Hann, S.& Troyer, C. (2020). Selective and accurate quantification of N-acetylglucosamine in biotechnological cell samples via GC–MS/MS and GC–TOFMS. *Anal. Chem.* 92, 4875.

Maky, M. A., Ishibashi, N., Zendo, T., Perez, R. H., Doud, J. R., Karmi, M.& Sonomoto, K. (2015). Enterocin F4-9, a novel O-linked glycosylated bacteriocin. *Appl. Environ. Microbiol.* 81, 4819.

Oberg, N., Zallot, R.& Gerlt, J. A. (2023). EFI-EST, EFI-GNT, and EFI-CGFP: Enzyme Function Initiative (EFI) web resource for genomic enzymology tools. *J. Mol. Biol.* 435, 168018.

Oman, T. J., Boettcher, J. M., Wang, H., Okalibe, X. N.& van der Donk, W. A. (2011). Sublancin is not a lantibiotic but an *S*-linked glycopeptide. *Nat. Chem. Biol.* 7, 78.

Stepper, J., Shastri, S., Loo, T. S., Preston, J. C., Novak, P., Man, P., Moore, C. H., Havlicek, V., Patchett, M. L.& Norris, G. E. (2011). Cysteine S-glycosylation, a new post-translational modification found in glycopeptide bacteriocins. *FEBS Lett.* 585, 645.
